# Nationwide genomic surveillance reveals the prevalence and evolution of honeybee viruses in China

**DOI:** 10.1186/s40168-022-01446-1

**Published:** 2023-01-11

**Authors:** Nannan Li, Cixiu Li, Tao Hu, Juan Li, Hong Zhou, Jingkai Ji, Jiangli Wu, Weipeng Kang, Edward C. Holmes, Weifeng Shi, Shufa Xu

**Affiliations:** 1 Institute of Apicultural Research, Chinese Academy of Agricultural Sciences, Beijing, 100193 China; 2 Shandong Provincial Hospital Affiliated to Shandong First Medical University, Jinan, China; 3 Key Laboratory of Emerging Infectious Diseases in Universities of Shandong, Department of Pathogen Biology, School of Clinical and Basic Medical Sciences, Shandong First Medical University & Shandong Academy of Medical Sciences, Taian, 271000 China; 4 Sydeny Institute for Infectious Diseases, School of Medical Sciences, The University of Sydney, Sydney, NSW 2006 Australia; 5 School of Public Health, Shandong First Medical University & Shandong Academy of Medical Sciences, Taian, 271000 China

**Keywords:** Honeybees, Ectoparasite mites, Meta-transcriptomics, Virus landscape, Evolution, Virus discovery

## Abstract

**Background:**

The economic and environmental value of honeybees has been severely challenged in recent years by the collapse of their colonies worldwide, often caused by outbreaks of infectious diseases. However, our understanding of the diversity, prevalence, and transmission of honeybee viruses is largely obscure due to a lack of large-scale and longitudinal genomic surveillance on a global scale.

**Results:**

We report the meta-transcriptomic sequencing of nearly 2000 samples of the two most important economic and widely maintained honeybee species, as well as an associated ectoparasite mite, collected across China during 2016–2019. We document the natural diversity and evolution of honeybee viruses in China, providing evidence that multiple viruses commonly co-circulate within individual bee colonies. We also expanded the genomic data for 12 important honeybee viruses and revealed novel genetic variants and lineages associated with China. We identified more than 23 novel viruses from the honeybee and mite viromes, with some exhibiting ongoing replication in their respective hosts. Together, these data provide additional support to the idea that mites are an important reservoir and spill-over host for honeybee viruses.

**Conclusions:**

Our data show that honeybee viruses are more widespread, prevalent, and genetically diverse than previously realized. The information provided is important in mitigating viral infectious diseases in honeybees, in turn helping to maintain sustainable productive agriculture on a global scale.

Video Abstract

**Supplementary Information:**

The online version contains supplementary material available at 10.1186/s40168-022-01446-1.

## Background

Honeybees provide pollination services for both a sustainable productive agriculture and the maintenance of a healthy non-agricultural ecosystem [1, 2]. However, the economic and environmental value of honeybees has been severely challenged in recent years by the collapse of honeybee colonies worldwide [3], likely associated with several biotic and abiotic factors [4–6], including parasitic mites (primarily *Varroa destructor*) and a number of pathogenetic viruses [7, 8]. To date, more than 70 viruses have been identified in honeybees and mites [9–11], with most seemingly associated with asymptomatic infection [11, 12] and only a tiny fraction causing disease with morphological, physiological, or behavioral changes [11, 13]. For example, the typical symptoms of deformed wing virus (DWV) infection include wing deformities, decreased body size, and discoloration [13]. A pale yellow appearance during larval development and larva failing to pupate are often associated with sacbrood virus (SBV) and black queen cell virus (BQCV) [13], while an inability to fly and abnormal trembling are associated with acute bee paralysis virus (ABPV), chronic bee paralysis virus (CBPV), and Israel acute paralysis virus (IAPV) infections [13]. These viruses normally persist as inapparent infections within the bee community. However, when they attain high virus titers or/and work synergistically with other abiotic and biotic stressors, they may lead to increased mortality and eventually the collapse of bee colonies [11].

Despite the obvious importance of documenting the virome of honeybees and their mites, genomic surveillance on a global scale has been sporadic. In particular, a lack of nationwide and longitudinal surveillance means that the prevalence, transmission, and host range of honeybee viruses in China are largely obscure [14–16]. Herein, we present a large-scale meta-transcriptomic survey of the virus landscape of the two most important economic and widely maintained honeybee species in China—the western honeybee (*Apis mellifera*) and the eastern honeybee (*Apis cerana*)—as well as the associated *Varroa destructor* ectoparasite mite. Most of the bees collected exhibited typical clinical signs, including overt symptoms of crawling or trembling, or were collected from colonies experiencing important worker bee losses. Our central aim was to reveal the prevalence, genetic diversity, and evolution of known and new viruses harbored by honeybees and mites across China. This information will help in the prevention and control of viral infectious diseases of honeybees both in China and globally.

## Results

### Composition and prevalence of known viruses in honeybees and mites

**Fig. 1:**
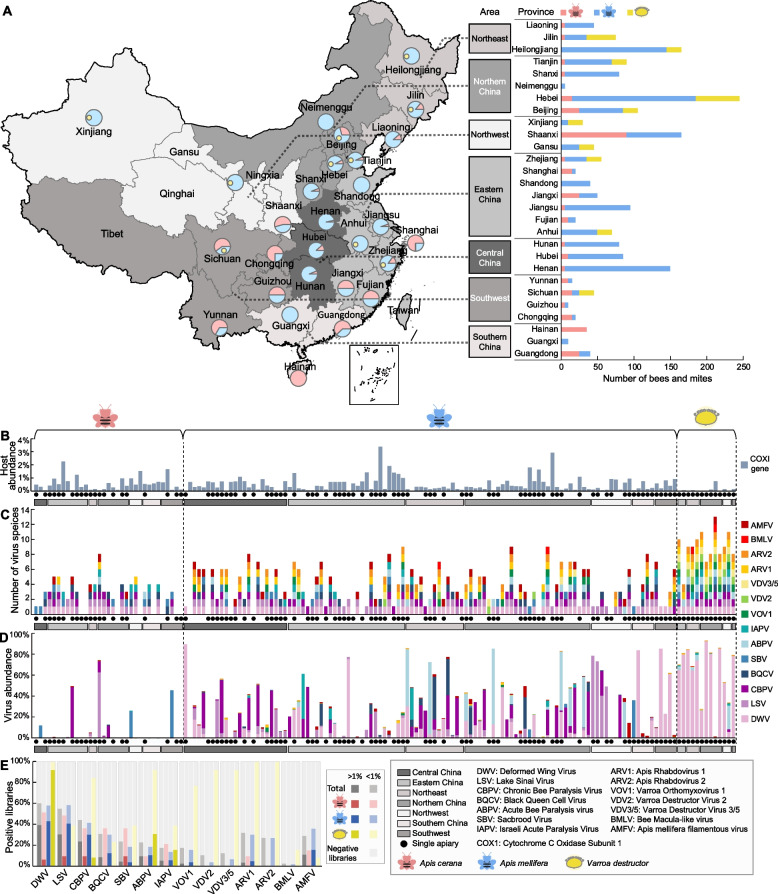
Identification of known honeybee viruses in China. **A** Sampling locations in China (left) and number of specimens surveyed in each host species in individual provinces (right), colored by host types. **B** Percentage of non-rRNA reads aligning to the honeybee and mite mitochondrial *COX1* reference sequences. **C** Number of known viral species identified in each library, colored by virus species. **D** Percentage of non-rRNA reads mapped to each RNA virus genomes in each library, colored by virus species. **E** Percentage of positive libraries for each virus, which was further subdivided at either >1% non-rRNA reads (dark color) or <1% non-rRNA reads (light color) within different host species

We performed meta-transcriptomic (i.e., total RNA) sequencing of 1290 western honeybees collected from 27 Chinese provinces, 335 eastern honeybees from 22 provinces, and 260 mites only from corresponding western honeybees from 10 provinces (Fig. 1A, Table S1) during 2016–2019. To better depict the prevalence and diversity of viruses discovered, we divided mainland China into seven regions: Northeast, Northern, Northwest, Eastern, Southwest, Southern, and Central China (Fig. 1A) in accordance with geographic location and climatic conditions [17]. The great majority of the samples were taken from colonies showing typical signs of disease, including overt symptoms of crawling or trembling. The remaining honeybees were collected from colonies that underwent important worker bee losses. These samples were pooled into 155 libraries based on the host species, collection location (province), and collection time (years), with the number of bees used per pool ranging from 5 to 60 (Table S1). Overall, total RNA sequencing generated 1.92 terabases of 150 bp paired-end reads, which were de novo assembled for virus screening (Table S1). The relative abundance of the host genes was assessed by estimating the abundance of cytochrome c oxidase subunit 1 (*COX1*) gene, ranging from 0.0006 to 3.3732% of total non-ribosomal RNA (non-rRNA) reads (Fig. 1B).

These transcriptome data allowed us to identify 14 known pathogenic honeybee viruses, with each library containing up to 13 viral species (Fig. 1C). Among them, ten belonged to positive-sense single-stranded (+ss) RNA viruses: deformed wing virus (DWV), Lake Sinai virus (LSV), chronic bee paralysis virus (CBPV), black queen cell virus (BQCV), sacbrood virus (SBV), acute bee paralysis virus (ABPV), Israeli acute paralysis virus (IAPV), Varroa destructor virus 2 (VDV2) [18], Varroa destructor virus 3/5 (VDV3/5) [2, 18], and bee macula-like virus (BMLV) [19]. Three belonged to negative-sense single-stranded (-ss) RNA viruses: Varroa orthomyxovirus 1 (VOV1) [2], Apis rhabdovirus 1 (ARV1) [10, 20], and Apis rhabdovirus 2 (ARV2) [10]. One belonged to double-stranded (ds) DNA viruses: *Apis mellifera* filamentous virus (AMFV). Eleven viruses—DWV, LSV, CBPV, BQCV, SBV, ABPV, IAPV, VOV1, ARV1, ARV2, and AMFV—were identified in all the seven Chinese regions (Fig. 1C). Hence, the majority of the 14 known viruses were prevalent throughout the country. Notably, these results provide the first evidence for the presence of VOV1, VDV2, VDV3/5, ARV1, ARV2, and BMLV in China. Of particular note, DWV and LSV were identified in more than 50% of the libraries, and DWV was detected in all mite libraries (Fig. 1E). In addition, aside from DWV, seven viruses—CBPV, IAPV, VOV1, VDV2, VDV3/5, ARV1, and ARV2—were identified in most (>75%) of the mite libraries (Fig. 1E).

We next estimated the proportion of viral reads to the total number of RNA reads (rRNA excluded): this ranged from 0.0001 to 92.60% across libraries (Fig. 1D). Viruses with high abundance (>1% of non-rRNA reads) included DWV (*n*=61), LSV (*n*=47), CBPV (*n*=37), SBV (*n*=7), ABPV (*n*=15), BQCV (*n*=13), IAPV (*n*=8), and AMFV (*n*=17) (Fig. 1E). Notably, DWV accounted for >1% of the non-rRNA reads in 92.31%, 43.12%, and 6.06% of the mite, western honeybee, and eastern honeybee libraries, respectively (Fig. 1E). The high abundance of viral reads in some honeybee libraries accords with previous reports of extremely high virus abundance in some invertebrate species [21, 22]. By investigating the 91 honeybee libraries collected from a single apiary, we found multiple viruses in almost 70% (63/91) of the honeybee colonies (Fig. 1C), with two to nine different viruses present. Hence, virus co-circulation is a common occurrence in individual honeybee colonies.

### Extensive diversity of known honeybee and mite viruses

**Fig. 2:**
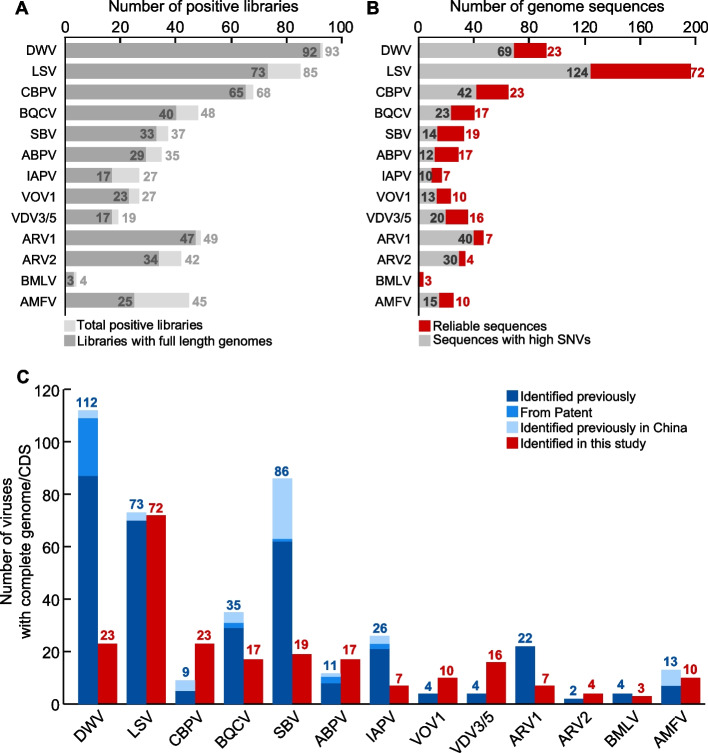
Summary of libraries and complete genomes of known honeybee and mite viruses obtained in this study. **A** Graphical representation of the total number of positive libraries (dark gray) and number of libraries with full length genomes (light gray) for each virus. **B** Graphical representation of number of credible sequences (red) and sequences with high SNVs (gray). **C** Graphical representation of the number of documented viruses in the NCBI GenBank sequence database (blue) and number of each virus species from this study (red)

With the exception of VDV2, full-length virus genomes (full-length DNA polymerase for AMFV) could be assembled from the positive libraries for the remaining 13 honeybee viruses (Fig. 2A), for which we were able to assemble a total of 615 consensus sequences (Fig. 2B). Among these, 218 were complete or near-complete genome sequences of high quality (i.e., the number of positions with heterogeneous bases divided by genome length <0.2%), including 23 DWV, 72 LSV, 23 CBPV, 17 BQCV, 19 SBV, 17 ABPV, 7 IAPV, 10 VOV1, 16 VDV3/5, 7 ARV1, 4 ARV2, and 3 BMLV, as well as 10 DNA polymerase gene sequence of AMFV (Fig. 2B). Together, these data greatly enhance the genomic resources of important honeybee viruses from public databases (as of 25th April, 2022), with the number of complete genomes of DWV increasing from 112 to 135, LSV from 73 to 145, CBPV from 9 to 32, BQCV from 35 to 52, SBV from 86 to 105, ABPV from 11 to 28, IAPV from 26 to 33, VOV1 from 4 to 14, VOV3/5 from 4 to 20, ARV1 from 22 to 29, ARV2 from 2 to 6, and BMLV from 4 to 7 (Fig. 2C).

**Fig. 3:**
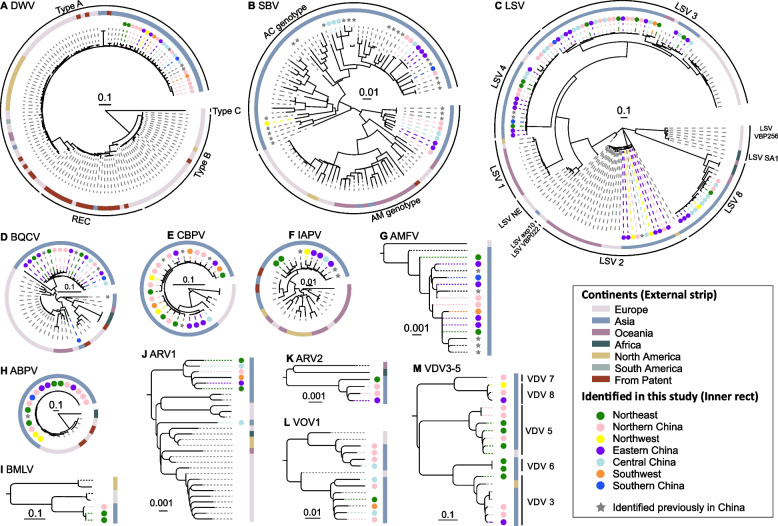
Intra-species diversity and phylogenetic relationships of known viruses in honeybees and mites. Viruses revealed in this study are colored by seven regions of China and highlighted with same color solid circle. Viruses identified previously in China are denoted by a solid grey star, whereas those representing the background phylogenetic diversity are not highlighted. All viruses are colored with an external strip according to the continents where they were identified. Genotype clusters are labeled on the trees for reference in the cases of DWV, SBV, LSV, and VDV3/5. The size of each circle was determined by the number of viruses used in each phylogenetic tree (See Supplementary Figs. S1-S10 for each tree in detail)

We next performed phylogenetic analyses of the reliable complete virus genomes (DNA polymerase gene sequence for AMFV) identified in this study and those available from GenBank (Fig. 3; phylogenies with more detailed information are shown in Figs. S1–S10). Global DWV has evolved into four major lineages: DWV-A, DWV-B, DWV-C, and several recombinant variants. DWV-C has been occasionally detected and until now was found only in the UK and Brazil [23]. DWV-B and the recombinant lineage mainly comprised sequences from Europe, although some from Africa, Asia, and South and North America also fell within this lineage (Fig. S1). DWV-A included sequences sampled worldwide, with the majority from Asia (Fig. S1). Notably, all DWV sequences documented in this study belonged to type A (Fig. 3A, Fig. S1). Furthermore, a detailed phylogenetic analysis of all Chinese DWV strains and selected references globally available from GenBank also revealed that all the reported DWV sequences from China belonged to type A (Fig. S11A).

Phylogenetic analyses revealed that the SBV isolates described in this study fell within both the AM and AC genotypes (Fig. 3B, Fig. S2). The AC genotype mainly comprised sequences from Asian countries, and the novel SBV strains from China were scattered within the AC lineage (Fig. 3B, Fig. S2). In contrast, the AM genotype of SBV comprised worldwide sequences, with the Chinese strains forming a distinct sub-clade that is paraphyletic to other sequences from the AM genotype (Fig. S2). In addition, all the Chinese sequences of the AM genotype were identified from *A. mellifera*, whereas those of the AC genotype were identified from both *A. mellifera* and *A. cerana*. Also of note was that a few SBV sequences from eastern and western honeybees were nearly identical (Fig. S2), suggesting potential cross-species transmission of SBV between the two honeybee species. Furthermore, the identical SBV sequences found in two provinces in great distances (i.e., Fujian and Hunan provinces) were most likely due to the practice of migratory beekeeping in China, which would potentially facilitated the long-distance spread of honeybee viruses.

LSV can be separated into various genotypes, including major genotypes such LSV1-LSV4 and LSV8 [24, 25], as well as several minor genotypes [25, 26]. Notably, all the major LSV genotypes comprise sequences sampled from diverse countries, suggesting their global prevalence. However, only one sequence of LSV4 has been previously identified in non-honeybee/non-mite insects [22], and one LSV2 sequence was identified in western honeybees in China [27]. Remarkably, phylogenetic analyses of the 72 complete LSV genomes assembled here revealed that they belonged to four genotypes: LSV2 (*n*=10), LSV3 (*n*=30), LSV4 (*n*=19), and LSV8 (*n*=13) (Fig. 3C, Fig. S3). Among the four genotypes, LSV3 and LSV4 were detected both in eastern and western honeybees, while LSV2 and LSV8 were only identified in western honeybees (Fig. 3C, Fig. S3). Importantly, LSV8 was identified in many sampling regions, with the exception of the southern and southwest regions (Fig. 3C, Fig. S3).

In the phylogenetic trees of BQCV (Fig. 3D, Fig. S4), CBPV (Fig. 3E, Fig. S5), IAPV (Fig. 3F, Fig. S6), and AMFV (Fig. 3G, Fig. S7), most of the newly identified sequences are grouped within previously described viruses from China. However, the novel Chinese ABPV sequences clustered with previously described viruses from Europe, while an ABPV sequence from Zimbabwe, Africa, formed a distinct sister clade (Fig. 3H, Fig. S8). No obvious geographic clustering was observed in these viruses (Fig. 3), although several grouped at the country or continent levels. Specifically, the variants of IAPV (Fig. 3F, Fig. S6) and AMFV (Fig. 3G, Fig. S7) identified in this study clustered with related strains from Asia, while CBPV (Fig. 3E, Fig. S5) identified here clustered with related sequences from China.

In the phylogenetic trees of BMLV (Fig. 3I, Fig. S7), ARV1 (Fig. 3J, Fig. S9), ARV2 (Fig. 3K, Fig. S9), and VDV3/5 (Fig. 3M, Fig. S10), some of the viruses newly described here formed separate and novel lineages, although this may reflect a lack of reference viruses. Apart from the recently described VDV3 and VDV5, we identified three new species clusters according to the threshold of 90% RdRp amino acid identity [28] in the VDV3/5 group. We therefore propose that these are named as Varroa destructor virus 6 (VDV6), Varroa destructor virus 7 (VDV7), and Varroa destructor virus 8 (VDV8), respectively (Fig. 3M, Fig. S10), although they were also found in *A. mellifera* samples. Hence, these data greatly expanded the documented genetic diversity of the pathogenic honeybee viruses, particularly ARV1, ARV2, VDV3/5, and BMLV.

### Discovery of novel RNA viruses in honeybees and their mites

Aside from known honeybee viruses, we also identified 23 novel genetically divergent virus genomes, including three negative-sense single-stranded RNA viruses and 20 positive-sense single-stranded RNA viruses (Table 1). Some double-stranded RNA viruses were similarly identified, although only with partial genome sequences due to low abundance (data not shown). These 23 viruses showed amino acid sequence similarity between 34.70 and 81.50% to the most conserved protein RNA-dependent RNA polymerase (RdRp) protein of their closest relatives (Table 1). All these virus genomes had greater than 20-fold genome coverage, with the highest reaching 32,978 fold (Table 1, Fig. S12). These novel viruses were named after the host genus (e.g., *Apis* and *Varroa*), followed by the name or category of the virus and a number if there is more than one virus (e.g., dicistrovirus 1). The relative abundance of the novel viruses exhibited substantial variation across libraries, ranging from 0.54 to 4277.53 RPKM (Table 1).

**Table 1:** Novel viruses discovered in this study

No.	Virus	ORF No.	Host	Genome length	Classification	Query domain	Identity (%)	Subject virus name	Sequencing depth (X)^a^	Abundance (RPKM)^a^	GenBank accession
**Positive-sense single-stranded RNA viruses**											
1	Apis picorna-like virus 1	2	*A. mellifera*	8530	*Marnaviridae*	RdRp	56.80	Beihai picorna-like virus 44	33	0.95	MZ822067
2	Apis picorna-like virus 2	2	*A. mellifera*	8588	*Marnaviridae*	RdRp	59.29	Shahe picorna-like virus 2	47	0.92	MZ822068
3	Varroa dicistrovirus 1	1	*V. destructor*	10319	Dicistro-related clade	RdRp	49.68	Weivirus-like virus sp	396	16.89	MZ822069
4	Apis dicistrovirus 2	2	*A. cerana*	8986	Dicistro-related clade	RdRp	54.98	Dicistroviridae sp.	44	1.01	MZ822070
5	Apis dicistrovirus 3	2	*A. mellifera*	9068	Dicistro-related clade	RdRp	63.01	Dicistroviridae sp.	334	12.13	MZ822071
6	Apis dicistrovirus 4	2	*A. mellifera*	8934	Dicistro-related clade	RdRp	64.21	Wenzhou picorna-like virus 34	92	2.04	MZ822072
7	Varroa dicistrovirus 2	2	*V. destructor*	8844	Dicistro-related clade	RdRp	51.67	Dicistroviridae sp.	414	14.90	MZ822073
8	Apis iflavirus 1	1	*A. mellifera*	10521	*Iflaviridae*	RdRp	53.18	Deformed wing virus	352	11.48	MZ822075
9	Apis iflavirus 2	1	*A. mellifera*	9356	*Iflaviridae*	RdRp	55.10	Picornavirales sp	22	0.73	MZ822076
10	Varroa picorna-like virus	1	*V. destructor*	7932	Picorna-related clade	RdRp	34.70	Wuhan house centipede virus 3	20	0.54	MZ822077
11	Apis picorna-like virus 3	1	*A. cerana*	10702	Picorna-related clade	RdRp	55.23	Blacklegged tick picorna-like virus 1	277	5.31	MZ822078
12	Apis picorna-like virus 4	1	*A. cerana* / *A. mellifera*	10033	Picorna-related clade	RdRp	53.66	Wuhan insect virus 12	32,978	4277.53	MZ822079- MZ822094
13	Apis picorna-like virus 5	1	*A. cerana*	10503	Picorna-related clade	RdRp	69.67	Solenopsis invicta virus 7	3,405	86.55	MZ822095
14	Apis picorna-like virus 6	2	*A. cerana*	11354	Picorna-related clade	RdRp	39.22	Hubei picorna-like virus 52	53	1.68	MZ822096
15	Apis nora virus 2	5	*A. mellifera*	10751	Picorna-related clade	RdRp	50.55	Helicoverpa armigera Nora virus	146	5.50	MZ822097
16	Apis polycipivirus	5	*A. cerana*	11714	*Polycipiviridae*	RdRp	68.76	Shuangao insect virus 4	54	1.22	MZ822098
17	Apis virga-like virus	3	*A. mellifera*	10401	Virga-related clade	RdRp	81.50	Allermuir Hill virus 2	436	7.25	MZ822100
18	Apis hypovirus 1	1	*A. mellifera*	13685	*Hypoviridae*	polyprotein	80.19	Fusarium sacchari hypovirus 1	142	2.58	MZ822101
19	Apis hypovirus 2	1	*A. mellifera*	11061	*Hypoviridae*	polyprotein	54.85	Wuhan insect virus 14	30	0.73	MZ822102
20	Apis hypovirus 3	1	*A. mellifera*	11285	*Hypoviridae*	polyprotein	53.98	Wuhan insect virus 14	396	4.34	MZ822103
**Negative-sense single-stranded RNA viruses**											
21	Apis rhabdovirus 3	6	*A. cerana*	13131	*Rhabdoviridae*	RdRp	45.11	Wuhan Fly Virus 2	89	3.92	MZ822104
22	Apis rhabdovirus 4	5	*A. cerana*	13489	*Rhabdoviridae*	RdRp	49.72	Diachasmimorpha longicaudata rhabdovirus	344	8.45	MZ822105
23	Apis rhabdovirus 5	5	*A. mellifera*	13489	*Rhabdoviridae*	RdRp	50.17	Diachasmimorpha longicaudata rhabdovirus	67	2.42	MZ822106- MZ822108

^a^The largest one is shown if detected in multiple libraries

*RPKM* reads per kilobase million

**Fig. 4:**
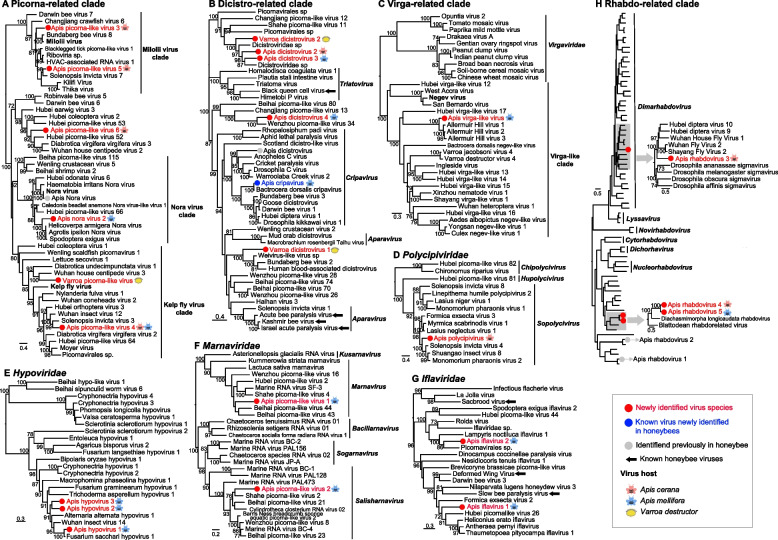
Evolutionary relationships of the novel viruses found in honeybees and mites. Each phylogenetic tree was estimated based on RdRp (RNA-dependent RNA polymerase) domains using a maximum likelihood method. Viruses identified in this study are marked in red/blue and highlighted with a red/blue solid circle, whereas those representing the background phylogenetic diversity are shown in black. Some viruses identified previously in honeybees are denoted in gray solid circle or with solid black arrow (See Supplementary Fig. S13 for detailed trees for panel h)

Phylogenetic analyses revealed that the 23 novel viruses described here fell within known viral families and clustered with invertebrate-associated viruses, with the majority likely associated with arthropods [21, 29]. Of the 20 positive-sense RNA viruses, 16 belonged to the order *Picornavirales* (Table 1), including six picorna-like viruses (Fig. 4A), five dicistro-related viruses (Fig. 4B), two *Marnaviridae* viruses (Fig. 4F), two *Iflaviridae* viruses (Fig. 4G), and one *Polycipiviridae* virus (Fig. 4D); three viruses belonged to the family *Hypoviridae* (Fig. 4E), and one belonged to the virga-like clade (Fig. 4C, Table 1). The three negative-sense RNA viruses grouped within the family *Rhabdoviridae* (order *Mononegavirales*) (Fig. 4H, Fig. S13, Table 1). The annotation of the virus genomes revealed similar conserved structures to their most closely related viruses (Fig. S12). Notably, eight of the viruses fell within two invertebrate-specific families— *Dicistroviridae* and the *Iflaviridae*—some of which have been demonstrated to infect honeybees (Fig. 4B and G), such as BQCV, ABPV, IAPV, DWV, SBPV, and SBV.

### Potential infectivity of novel RNA viruses in honeybees

Because active replication of RNA viruses will often elicit RNA interference (RNAi) pathways in insects and boost the production of antiviral small RNAs [1, 30, 31], we screened for the presence of small RNAs in honeybees and mites. By performing tagged RT-PCR (Table S2), we also examined the presence of the counterpart complementary strands of 16 viral positive- or negative-sense RNA: these are viral replication intermediates and hence an indicator of ongoing replication of RNA virus [32].

**Fig. 5:**
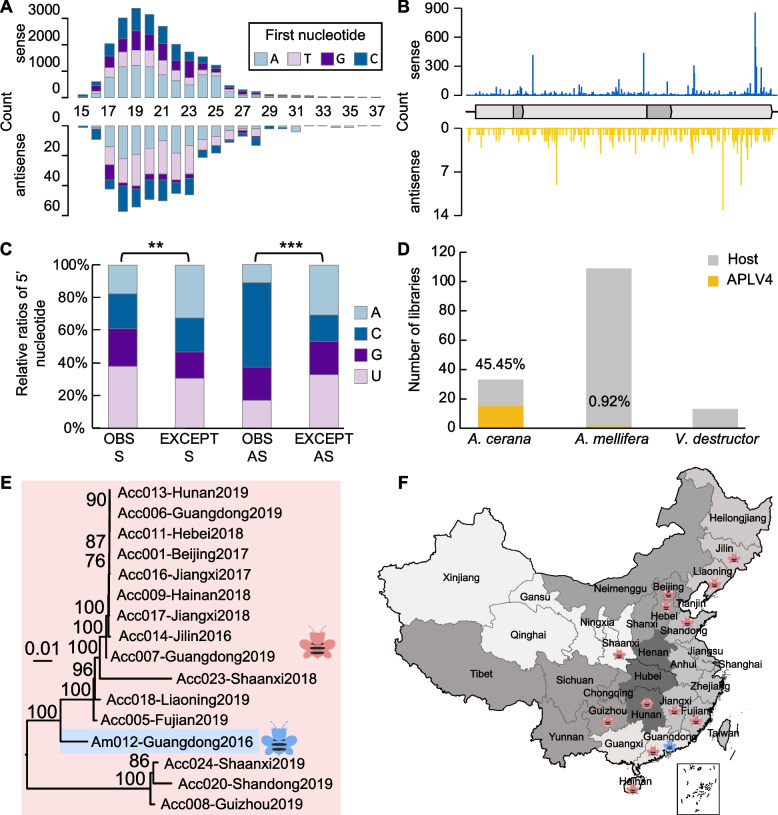
Analysis of small RNAs and variants of Apis picorna-like virus 4. **A** Size distributions (15 to 37 nt) and 5’-nucleotide compositions of small RNAs arising from APLV4. **B** Mapping of 20- to 23-nt-long viral RNAs to the genome of APLV4. **C** Observed 5’ nucleotide (Obs) compared with that expected (Exp) from the base compositions of the viral genomes for APLV4. Sense (S, high-expressed strand) and antisense (AS, low-expressed strand) reads were compared by using a chi-squared test. **p <0.01; ***p < 0.001. **D** Percentage of positive libraries for APLV4 within different host species. **E** Phylogenetic tree of APLV4 complete genomes identified in honeybees collected from different regions of China from different years. **F** Chinese provinces in which APLV4 was detected. Eastern and western honeybees are colored in red and blue, respectively

We prepared small RNA libraries using the same samples used for meta-transcriptomic sequencing and with novel viruses, generating 16.67 billion bases of 50 bp single-end reads (Table S3). Non-host small RNA reads were aligned to the genomes of all novel viruses (Fig. S12, Table S3). From this, we identified highly abundant small RNAs mapping to Apis picorna-like virus 4 (+ss RNA virus) (APLV4) (Fig. S12, Table S3), with a size distribution of 17 to 25 nt in both sense and antisense orientations (Fig. 5A), targeting the “hot spots” of the viral genome or anti-genome (Fig. 5B). These are typical signatures of dicer-produced antiviral RNAs [10, 33, 34]. Furthermore, as the antiviral immune response is mediated by RNA-binding proteins, which shows a 5’-terminus nucleotide preference in small RNA [33, 34], we also determined the base composition of 5’-terminus of small RNA. This revealed a significant preference for the 5’-end U in the high-expressed strand (sense) and for the 5’-end C of the low-expressed strand (antisense) (Fig. 5C), consistent with the small RNA strand selection in flies [33]. Meanwhile, tagged RT-PCR also identified the antisense segment of the APLV4 genome (Fig. S14). Collectively, our data strongly indicated that bees have an active antiviral immune response against APLV4 and APLV4 can replicate in bees.

It was notable that APLV4 was detected in both eastern and western honeybees, presenting in 45.45% (15/33) and 0.92% (1/109) of their libraries, respectively (Fig. 5D). The APLV4 identified from individual libraries carried unique single-nucleotide polymorphisms, exhibiting 87.86 to 100% nucleotide identity and 94.20 to 100% amino acid similarity to each other (Table S4). Sequences from each library formed distinct phylogenetic clusters, suggesting that they are not due to cross-sample contamination (Fig. 5E). Importantly, APLV4 was detected in samples collected each year between 2016 and 2019 (Fig. 5E) and in provinces from all seven Chinese regions (Fig. 5F), suggesting its long-term circulation across China.

Finally, eight additional novel viruses were mapped with small RNAs (Fig. S12, Table S3), including Varroa dicistrovirus 1-2, Varroa picorna-like virus, Apis picorna-like virus 5, Apis nora virus 2, Apis iflavirus 1, and Apis rhabdovirus 3-4. However, only a low abundance of small RNA reads mapped to these viruses. Meanwhile, tagged RT-PCRs on three viruses (Apis dicistrovirus 3, Apis iflavirus 1, and Apis rhabdovirus 4) were positive (Fig. S14). Further investigation of the potential infectivity of these viruses is required.

### Assessing the impact of host biology on virome composition

**Fig. 6:**
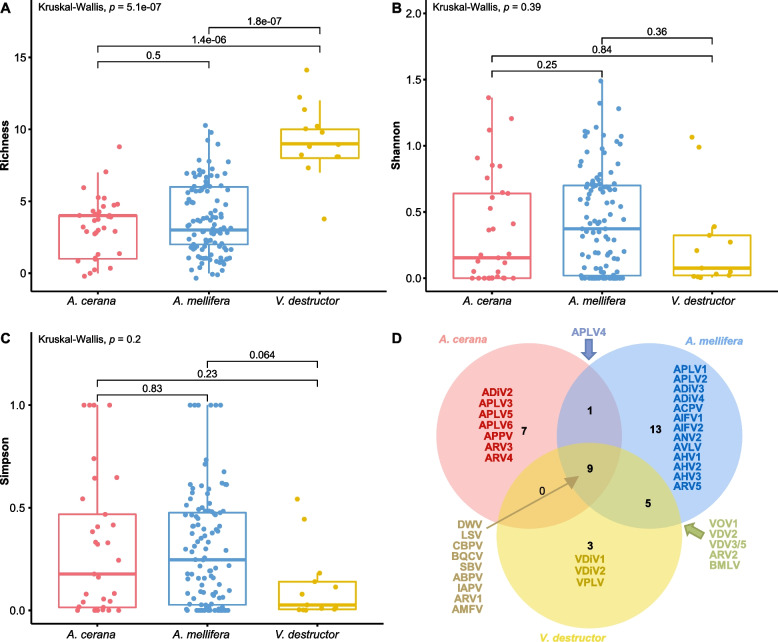
Comparison of virome composition between two species of honeybee and mite communities. **A** Virome richness, **B** Shannon, **C** Simpson, and **D** Venn diagrams showing the differences of virome composition between different communities

We next examined the possible association between eastern and western honeybee communities, using alpha diversity (within each community, measured by observed richness, Shannon index, and Simpson index), and beta diversity (between communities). There was no significant difference (*p* > 0.05) in alpha diversity between the two honeybee groups according to any of the above methods (Fig. 6A–C), indicating that honeybee species might not influence the virome structure. However, the greatest virus richness was found in the mite group with a median of 9, significantly higher than those of both eastern and western honeybee groups (medians of 4 and 3, respectively) (Fig. 6A), while there were no significant differences among all groups for the Shannon and Simpson indexes (Fig. 6B, C).

The virome compositions among the three groups were further displayed using Venn diagrams. Excluding the nine viruses shared by the three groups, only one virus (APLV4) was shared by eastern and western honeybees, five viruses (VOV1, VDV2, VDV3/5, ARV2, and BMLV) were shared by western honeybees and mites, while no additional viruses were shared by eastern honeybees and mites (Fig. 6D). The virome composition (mainly novel viruses) varied among groups (Fig. 6D). For instance, seven viruses were only found in eastern honeybees, 13 viruses only in western honeybees, and three only in mites (Fig. 6D). In addition, PCoA plots based on the Bray–Curtis distance matrices revealed that the samples from different groups did not form distinct clusters (Fig. S15).

## Discussion

We present the first large-scale and longitudinal viral genomic surveillance of the two most important economic and widely maintained honeybee species—the western honeybee (*A. mellifera*) and the eastern honeybee (*A. cerana*)—as well as the related ectoparasite mite *Varroa destructor* across China. Analysis of the transcriptomes of nearly 2000 samples revealed an expanded geographic distribution and genetic diversity of the important honeybee viruses in China and provided evidence that the co-circulation of multiple viruses in single a honeybee colony is commonplace. In particular, we provide the first evidence that most known honeybee viruses are prevalent in China, highlighting the serious challenges posed by viral infectious diseases and strongly suggesting that the large-scale movement of beekeepers and their bees across mainland China provides viral connectivity between geographically distinct localities, potentially facilitating the spread of honeybee viruses.

DWV is the most widespread and well-studied bee virus globally and has been detected in at least 65 arthropod species [35]. DWV infection causes symptoms in adult honeybees, including wing malformations, shortened abdomens, and even severely reduced longevity leading to colony collapse disorder particularly during the winter months in the northern hemisphere [8, 35]. The DWV complex consists of three closely related variants (DWV-A, DWV-B, and DWV-C), as well as various recombinants [36]. DWV-A was initially the dominant genotype in several western countries and areas, such as the Hawaiian Islands [37], Europe, and South Africa [38]. However, a shift from DWV-A to DWV-B has occurred over the past 20 years, and DWV-B has almost replaced DWV-A as the dominant type in western countries [37]. In contrast, in Asian countries such as China, Japan [39], South Korea, and India, as well as in Oceanic regions (Fig. S11B), DWV-A still predominates in honeybee communities. These results suggest that DWV-A might be solely responsible for current DWV-related colony losses in China. Accordingly, the continuous monitoring of DWV is warranted in China.

SBV is also an important honeybee pathogen causing failure in pupation and death in both larvae and adult honeybees [40]. The virus has been divided into two major groups—the AM and AC genotypes [41, 42]. The AM genotype is composed of SBV strains identified in western honeybees worldwide, and the novel Chinese sequences identified here were also found in western honeybees, forming a distinct clade indicative of some isolated evolution. In contrast, the novel Chinese sequences of the AC genotype were found in both the eastern and western honeybees and clustered with these previously described in China. It is notable that the distribution of the SBV viruses of the AC genotype, whether in eastern or western honeybees, is mainly restricted to Asia [11]. In addition, cross-species transmission events between *A. mellifera* and *A. cerana* were observed within the AC genotype [43], which might represent a high-risk factor for SBV recombination, in turn potentially leading to a more pathogenic variant.

Another prevalent virus complex is associated with acute paralysis (named the AKI-complex) [40] in honeybees, comprising three viruses: ABPV, Kashmir bee virus (KBV), and IAPV. These viruses belong to the same genus *Aparavirus* (family *Dicistroviridae*), sharing close genetic relationships and several biological characteristics [11, 44]. These viruses are infamous for their close association with the *V*arroa mite, believed to serve as both a vector and an activator of the AKI-complex in honeybee colonies [13], and leading to multiple paralysis symptoms at elevated titers as well as the decline and collapse of honeybee colonies, especially in those infested with parasitic mites. However, in contrast to ABPV and IAPV which are present in both species of honeybee and mites, KBV was not detected in this study, providing additional evidence that it is absent from China [14, 16, 45]. Similarly, slow bee paralysis virus (SBPV), associated with colony collapse and efficiently transmitted by mites [46], was not detected here, and hence may be absent or very rare in China.

We identified a high prevalence of LSV (positive in >50% libraries) in honeybees, including the first description of LSV3 and LSV8 in China. LSVs have a broad range of hosts including honeybees, ants, solitary bees, bumble bees and hornets [47], which seem to share similar ecological niche and frequently interact, potentially facilitating cross-species virus transmission among them. In addition, super-infections with up to three distinctive clades were present in a single honeybee [48], potentially enabling intra-species recombination among different LSV groups and increasing its genetic diversity. Importantly, however, the pathogenicity of LSV is not well understood, and LSV may be a co-factor of collapsing honeybee colonies [49], as it was present with high abundance in both diseased and healthy honeybee colonies [24].

Our results constitute the first evidence for the presence of VOV1, VDV2, VDV3/5, ARV1, ARV2, and BMLV in China. Notably, these viruses were detected in both western honeybees and *V*arroa mites, reflecting the expanding pathogen spectrum of honeybee viruses. Detection of the presence of antisense strand viral RNA suggests that some of these viruses, such as VOV1, ARV1, ARV2, and BMLV, could replicate in both honeybee and mites, while VDV2 and VDV3/5 were restricted to mites. However, whether these viruses are pathologic and what influence these viruses have on colony health is unknown. With the exception of ARV1, all these newly identified viruses in China were not detected in eastern honeybees, although this could also reflect insufficient sampling.

Another key result from our study was that mites harbored significantly higher virus diversity than the western honeybees they were sampled from. Although both Shannon and Simpson indexes showed no difference in those two communities, the alpha diversity measured using observed richness suggested that virome composition differs between western honeybees and mites. Based on the comparison of the paired western honeybee and mite samples collected from the same apiary (data not shown), all western honeybee viruses were detected in mites, while some viruses present in mites were not found in western honeybees. Combined, these data are indicative of frequent virus transfers between mites and western honeybees such that mites could act as vectors for western honeybee viruses. Furthermore, we identified three novel genetic variants related to VDV3/5 in both mite and *A. mellifera* samples, highlighting the extent of genetic diversity that can be carried by these ectoparasites.

Overall, we identified 23 novel viruses, one of which clearly merits additional investigation as a potential pathogen (APLV4) based on the following: (i) typical signatures of antiviral immune response in honeybees, (ii) the identification of the negative-sense genomic segment, and (iii) the presence of sequences with unique single nucleotide polymorphisms. APLV4 was detected in 15 eastern honeybee libraries across China with particularly high prevalence, as well as in one western honeybee library, and it may be pathogenic to at least eastern honeybees. However, additional genomic surveillance and studies of the pathogenicity of APLV4 in both eastern and western honeybees are clearly warranted. In addition to APLV4, both small RNA data and tagged RT-PCR results have revealed the potential infectivity of a number of other viruses, which similarly merit further investigation.

The small RNA patterns of APLV4 in honeybees showed typical signatures of Dicer-mediated degradation, with 17 to 25 nt long siRNA duplexes peaking at 19 nt. However, a hallmark of host RNAi response in Drosophila is a small RNA distribution with a peak at 21 nt [50]. In addition, the small RNA reads of the same virus presented in different hosts showed markedly different characteristics, reflected by the distinct length distribution and the peak [10]. Hence, we believe that the small RNA profiles of viruses might vary in different arthropod hosts, across different viruses, and even probably with the stage of infections. Furthermore, the low abundance of some novel viruses discovered in this study may partly reflect the effective host RNAi response during the stage of infection. It is also possible that viruses with low abundance may in fact be derived from gut microflora or parasites in the hosts [21].

More broadly, our data have greatly expanded the genomic resources of important honeybee viruses, revealing greater diversity than previously realized. As such, our study provides invaluable information to develop novel or update available detection assays (i.e., PCR primers) of honeybee viruses. However, given the drawbacks of pooling of multiple individual samples, it would be important to sample and sequence individual honeybees. This would provide valuable information on viral prevalence within honeybee populations, and also the intra-host single nucleotide variations (SNVs) of honeybee viruses. In addition, bacterial, and eukaryotic microbes carried by honeybees might influence virus carriage, transmission, and pathogenesis, which also warrants further investigation.

## Conclusions

Our nationwide and longitudinal study highlights the wide diversity and high prevalence of known pathogenic honeybee viruses in the two major honeybee species throughout China. Although our study sheds light on the serious challenges that viruses have imposed on honeybees, additional research is required to determine whether the virus transmission is associated with the annual mass human-mediated movement of honeybees from southern to northern China, as well as the viral connectivity between migrated western and eastern honeybees. The genomic data presented here will be of importance to the prevention and control of viral infectious diseases in honeybees and help maintain the balance between bee ecology and the beekeeping industry both in China and globally.

## Methods

### Sample collection

This study was based on the analysis of 155 libraries of honeybee and mite samples obtained from 326 honeybee colonies in various locations in China between 2016 and 2019 (Table S1). The mainland of China was divided into seven regions–Northeast, Northern, Northwest, Eastern, Southwest, Southern, and Central China—based on geographical location and climatic conditions. Overall, 67 *A. cerana* colonies were collected in 22 provinces, including four from central China, 12 from eastern China, two from northeast China, 10 from northern China, 18 from northwest China, 12 from southern China, and 9 from southwest China. Similarly, 259 *A. mellifera* colonies were collected in 27 provinces, including 59 colonies from central China, 50 from eastern China, 43 from northeast China, 75 from northern China, 22 from northwest China, five from southern China, and five from southwest China. Samples were collected in two ways: (1) diseased bees were sent by beekeepers to our lab (one of the surveillance centers of bee diseases of the Ministry of Agriculture, China), and (2) samples suspected to have a viral infection were collected during our routine surveillance of some apiaries. Twenty *Varroa destructor* mites were collected from 13 western honeybee colonies, including those described above, of which two colonies were from eastern China, three from northeast China, five from northern China, two from northwest China, and one from southwest China. None of these colonies had been treated with acaricide. All samples were captured alive and then transported on dry ice from the field to the laboratory, and store at −80°C until further processing.

### Sample processing

Five bees from each colony were first grouped as a unit. These units were further pooled into 109 western honeybee pools and 33 eastern honeybee pools based on bee species, sampling location, and collection time. Twenty varroa mites from the same honeybee colony were similarly grouped together as a pool. These samples were frozen in liquid nitrogen and then ground into fine powder using a mortar and a pestle. Total RNA was extracted using TRIzol reagent (Thermo Fisher) with about 50mg of powder from each pool. RNA integrity and quantity were measured using the RNA Nano 6000 Assay Kit of the Bioanalyzer 2100 system (Agilent Technologies). For all libraries, we used the TruSeq stranded total RNA paired-end libraries protocol (Illumina). Briefly, following DNase I digestion, the ribosomal RNA was depleted from total RNA using the Ribo-Zero plus kit (Illumina) following the manufacturer’s instructions. The remaining RNA was fragmented, reverse-transcribed, ends adenylated and adaptor-ligated. After purification of cDNA, amplification was performed using PCR. Paired-end (150bp) sequencing of each RNA library was performed on the HiSeq 2000 platform (Illumina). All library preparation and sequencing were performed by Novogene Company (Beijing, China).

### Sequence assembly and target virus discovery

For each library, sequencing reads were first adapter trimmed and low quality/complexity filtered using Fastp v0.19. (default settings) [51], with ribosomal (r)RNA reads removed using SortMeRNA(v 2.1b) [52] mapping on the SILVA_132 rRNA database. The remaining reads were assembled de novo using the Trinity (v2.5.1) program [53] with default parameter settings. The assembled contigs were compared against the non-redundant nucleotide (nt) and non-redundant protein (nr) databases (downloaded and built on 2021/02/22) using Blastn v2.10.0+ [54] and Diamond v.0.9.32 [55], respectively, with *e* value thresholds of 1×10^-10^ and 1×10^-5^ to obtain high sensitivity at a low false-positive rate. For virus contigs associated with known honeybee and mite viruses, reads were directly mapped to the sequence of a close relative using Bowtie2 v2.3.5.1 [56], and a consensus genome was obtained from the mapped reads as the final sequence, with thresholds of the consensus ≥90% at all sites and the number of degenerate bases ≤0.2% of the genome length. Ambiguous bases were determined by consensus threshold setting (90%) for IUPAC ambiguity codes in Geneious prime 2019.2.3 (Biomatters Ltd., Auckland, New Zealand), which are counted as fractional support for each nucleotide.

As the barcode hopping rate is usually between 0.01 and 0.1% [57], to exclude the reporting of false-positive results due to cross-contamination and index-hopping during sequencing, a “true” positive library had to meet the following criteria: (i) the virus was detected in less than half of the libraries sequenced on the same sequencing lane and (ii) the read abundance of the virus was more than 0.1% of that representing the highest count for that virus among the other libraries. Furthermore, in the case of previously documented honeybee viruses that commonly share >99% nucleotide similarity with each other, genome sequences with high ratio of SNVs (described above) were excluded from the alignment and phylogenetic analyses as a single genome sequence, but rather analyzed as polymorphic sites within each virus species.

As each pool includes multiple individuals, cases with multiple variants of one virus species could exist. For virus species LSV, VDV2, and VDV3/5 that had multiple variants in the same pool, we employed an “iterative assembly” strategy to separate the sequences with variable sites. For example, all LSV-associated contigs were extracted from the initial assembled sequences, followed by read mapping using Bowtie2. All mapped reads were extracted as a sub-library and assembled de novo into contigs. LSV-associated contigs were extracted again and merged by identifying unassembled overlaps between neighboring contigs using Geneious Prime. This step usually provides one or two LSV genomes with full-length sequences. Confirmation of LSV genomes was performed by read mapping using reads in the sub-library, with the final majority consensus sequences determined from the final assembly of mapped reads using Geneious Prime. The unmapped reads in the sub-library were extracted as a second sub-library and assembled de novo again into contigs. These steps were repeated until no reads left in the sub-library or no more complete LSV genomes were identified.

Oxford Nanopore sequencing was used to confirm the entire genome of virus species with multiple variants. Six pools with multiple virus variants were selected (Table S5) and the original RNA samples were used for sequencing. First, cDNA was made with random primers using the PrimeScriptTM RT Reagent Kit (Perfect Real Time) (Takara) following the manufacturer’s instructions, followed by randomly amplification using 2×S6 PCR MasterMix(FL). Amplified cDNA integrity was purified using BluePippin gel cassettes and quantified using a Qubit high-sensitivity DNA kit (Thermo Fisher). Sequencing libraries were prepared using the Ligation Sequencing kit (SQK-LSK109) and barcoded individually using the Ligation Sequencing kit (EXP-NBD104) (Oxford Nanopore Technologies). Libraries were sequenced on R9.4.1 flow cells on PromethION device (Oxford Nanopore Technologies). All library preparation and sequencing were performed by the BENAGEN Company (Wuhan, China). Nanopore reads were base called using Guppy v4.0.23 (Oxford Nanopore Technologies), and output fastq files were then adaptor trimmed and quality filtered using NanoFilt v2.6.0 [58]. The remaining reads were mapped against the reference sequence using Minimap2 v2.17-r974-dirty [59] and reference virus-associated reads were extracted and then assembled into contigs using Canu v2.2.1 [60]. Clean Illumina sequencing reads from the same pool were then mapped on those assembled contigs for confirmation.

For the novel viruses, the viral sequences were merged by identifying unassembled overlaps between neighboring contigs or within a scaffold [53] using Geneious Prime, followed by iterative read mapping for further extension of the genome at both ends. We used RT-PCR and Sanger sequencing to fill the gaps on incomplete genomes, mapping reads back to the full-length genome to confirm the final assembly results. Prediction of the potential genome length and conserved domains in each virus was initially based on those from related reference virus genomes. Open reading frames were predicted using the online tool ORFfinder and functional predictions of conserved domains within encoded proteins were made against the Conserved domain database (CDD) and then against nor-redundant protein database at NCBI.

### Inferring virus phylogenetic history

In the case of previously known viruses, all complete or near complete reference sequences present in the current NCBI database were included as background. In the case of novel viruses, we retrieved the most conserved domain (RNA-dependent RNA polymerase; RdRp) of their corresponding homologs of reference viruses from NCBI. We used the MAFFT v7.407 program [61] to align each group of sequences using the E-INS-I algorithm, followed by the subsequent removal of all ambiguously aligned regions using TrimAl v1.4 [62]. Phylogenetic trees were inferred using the maximum likelihood method implemented in IQ-TREE v1.6.12 [63], employing the best-fit substitution model for each data set identified by IQ-TREE.

### Small RNA library preparation and sequencing

A total amount of 3-μg total RNA per sample was used to generate a small RNA sequencing libraries using NEBNext Multiplex Small RNA Library Prep Set for Illumina (NEB, USA.) following the manufacturer’s protocol. Index codes were added to attribute sequences to each sample. Library quality was assessed on the Agilent 2100 Bioanalyzer (Agilent Technologies). After the clustering of the index-coded samples, performed on the cBot Cluster Generation system using TruSeq SR Cluster Kit v3-cBot-HS (Illumia), 50bp single-end sequencing of each small RNA library was performed on the Illumina Hiseq 2500/2000 platform. All library preparation and sequencing were performed by the Novegene Company (Beijing, China).

### Small RNA analysis

Small RNA reads were first adapter trimmed and low quality/complexity filtered using cutadapt (default settings) program [64], and the rest reads were mapped to the corresponding host reference genomes using Bowtie2. The unmapped reads of each library were subsequently mapped to the corresponding 23 novel virus genomes, allowing one nucleotide mismatch, which were exported as BAM files for further analysis. Nucleotide size, size distribution, genome position, and base composition of small RNA reads for both sense and antisense strand were analyzed using in-house Perl and Python scripts, available at “https://github.com/lihannan/Bee-Virome.”

### Replication assay

Tagged RT-PCR was performed to detect complementary strand (replicative intermediate) RNA, as it was regarded as an indicator of ongoing replication of RNA virus. Different tagged primers were synthesized based on positive-strand and negative-strand RNA virus separately and used in the detection of the complementary strand for each corresponding novel virus described in this study. The reverse transcription (RT) reaction was performed at 50°C for 30 min in the presence of the tagged primers (Supplementary Table 3) using PrimeScript^TM^ RT Reagent Kit (Perfect Real Time) (Takara). The RT product was used to conduct PCR assays using the specific primers and the paired tagged primers as above. The reaction mixtures were first denatured at 98°C for 30 s, 35 cycles at 98°C for 10 s, then 52°C for 30 s, 72°C for 30 s, and a final extension at 72°C for 5 min. The identity of the PCR product was confirmed by Sanger sequencing.

### Revealing virome abundance and statistical analysis

The relative abundance of a host reference gene (COX1) and total viral RNA was presented as the proportion of the total non-ribosomal RNA. A virus was considered to be of high abundance if it represented >0.1% of total ribosomal-depleted RNA reads in the library. The relative abundance of each virus was also presented as the number of Reads per kilobase per million mapped reads (RPKM), calculated as “mapped reads/(viral-length/1000*total reads/one million).” Bowtie2 was used to align the reads to each novel virus genome and SAMtools [65] was used to compute the percent of reads mapped and coverage depth. Statistical analysis of viral diversity and abundance were performed using the *t* test or Wilcoxon test based on results of normal distribution test (shapiro.test) in the ggpubr package and were plotted using ggplot2 package in RStudio version 4.1.2. The observed richness, Shannon index and Simpson index (i.e., Alpha diversity) were measured for each library using modified Rhea script sets [66] and compared between eastern, western honeybee, and mite groups using the Kruskal-Wallis rank sum test. Principal coordinate analysis (PCoA) (i.e., Beta diversity) was performed based on Bray-Curtis dissimilarity matrix using the Vegan package [67].

## Supplementary Information

**Additional file 1: Table S1.** Host and geographic information and data output for each pool. **Table S2.** Tagged primer pairs used in the present study and RT-PCR results. **Table S3.** Small RNA libraries and data generated. **Table S4.** Intra-species nucleotide (nt) and amino acid (aa) similarity (%) of APLV4. **Table S5.** Summary of results from Nanopore sequencing. **Table S6.** Summary of the bioinformatics resources used in this study.

**Additional file 2: Figure S1.** Maximum likelihood phylogeny of DWV. **Figure S2.** Maximum likelihood phylogeny of SBV. **Figure S3.** Maximum likelihood phylogeny of LSV. **Figure S4.** Maximum likelihood phylogeny of BQCV. **Figure S5.** Maximum likelihood phylogeny of CBPV. **Figure S6.** Maximum likelihood phylogeny of IAPV. **Figure S7.** Maximum likelihood phylogeny of BMLV-AMFV. **Figure S8.** Maximum likelihood phylogeny of ABPV. **Figure S9.** Maximum likelihood phylogeny of ARV1-ARV2. **Figure S10.** Maximum likelihood phylogeny of VOV1-VDV3/5. **Figure S11.** Intra-species diversity and global distribution of DWV. **Figure S12.** Summary of genomic features of the novel viruses identified in this study. **Figure S13.** Maximum likelihood phylogeny of the rhabdoviruses. **Figure S14.** Tagged RT-PCR results on four viruses. **Figure S15.** Beta diversity analysis of the viromic composition among libraries.

## Data Availability

All sequence reads generated in this study have been uploaded onto the NCBI Sequence Read Achieve (SRA) database under the BioProject accession PRJNA706851. All novel and known virus genome sequences generated in this study have been deposited at NCBI/GenBank under the accession numbers MZ822067-MZ822108, MZ821771-MZ822058, and MZ826697.
