# Supplementary material for: Nationwide genomic surveillance reveals the prevalence and evolution of honeybee viruses in China
Source: Microbiome. 2023 Jan 11;11:6. doi: 10.1186/s40168-022-01446-1 (PMC9832778; doi:10.1186/s40168-022-01446-1)
Supplement: Supplementary file 3 — Additional file 2: Figure S1. Maximum likelihood phylogeny of DWV. Figure S2. Maximum likelihood phylogeny of SBV. Figure S3. Maximum likelihood phylogeny of LSV. Figure S4. Maximum likelihood phylogeny of BQCV. Figure S5. Maximum likelihood phylogeny of CBPV. Figure S6. Maximum likelihood phylogeny of IAPV. Figure S7. Maximum likelihood phylogeny of BMLV-AMFV. Figure S8. Maximum likelihood phylogeny of ABPV. Figure S9. Maximum likelihood phylogeny of ARV1-ARV2. Figure S10. Maximum likelihood phylogeny of VOV1-VDV3/5. Figure S11. Intra-species diversity and global distribution of DWV. Figure S12. Summary of genomic features of the novel viruses identified in this study. Figure S13. Maximum likelihood phylogeny of the rhabdoviruses. Figure S14. Tagged RT-PCR results on four viruses. Figure S15. Beta diversity analysis of the viromic composition among libraries. [file 40168_2022_1446_MOESM2_ESM.pdf]

Supplementary Figures

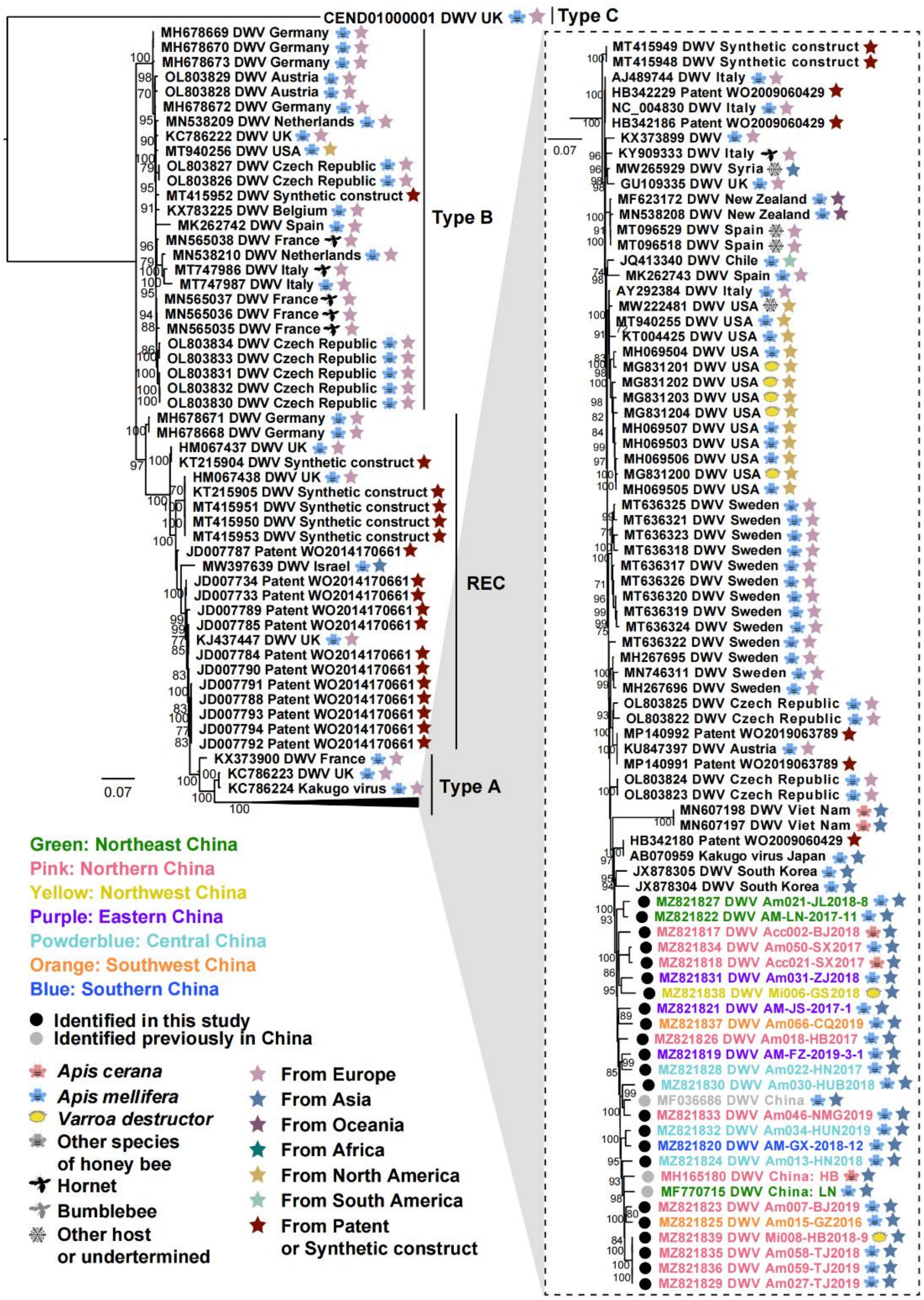

Figure S1. Maximum likelihood phylogeny of DWV. The names of the viruses are marked in different colours based on the geographical locations of their host in China. Viruses identified in this study are distinguished from previously documented viruses with black solid circles at the beginning of their names. The silhouetted animal host taxa and the colored star symbol denoting the continental of sampling of the related viruses are also shown.

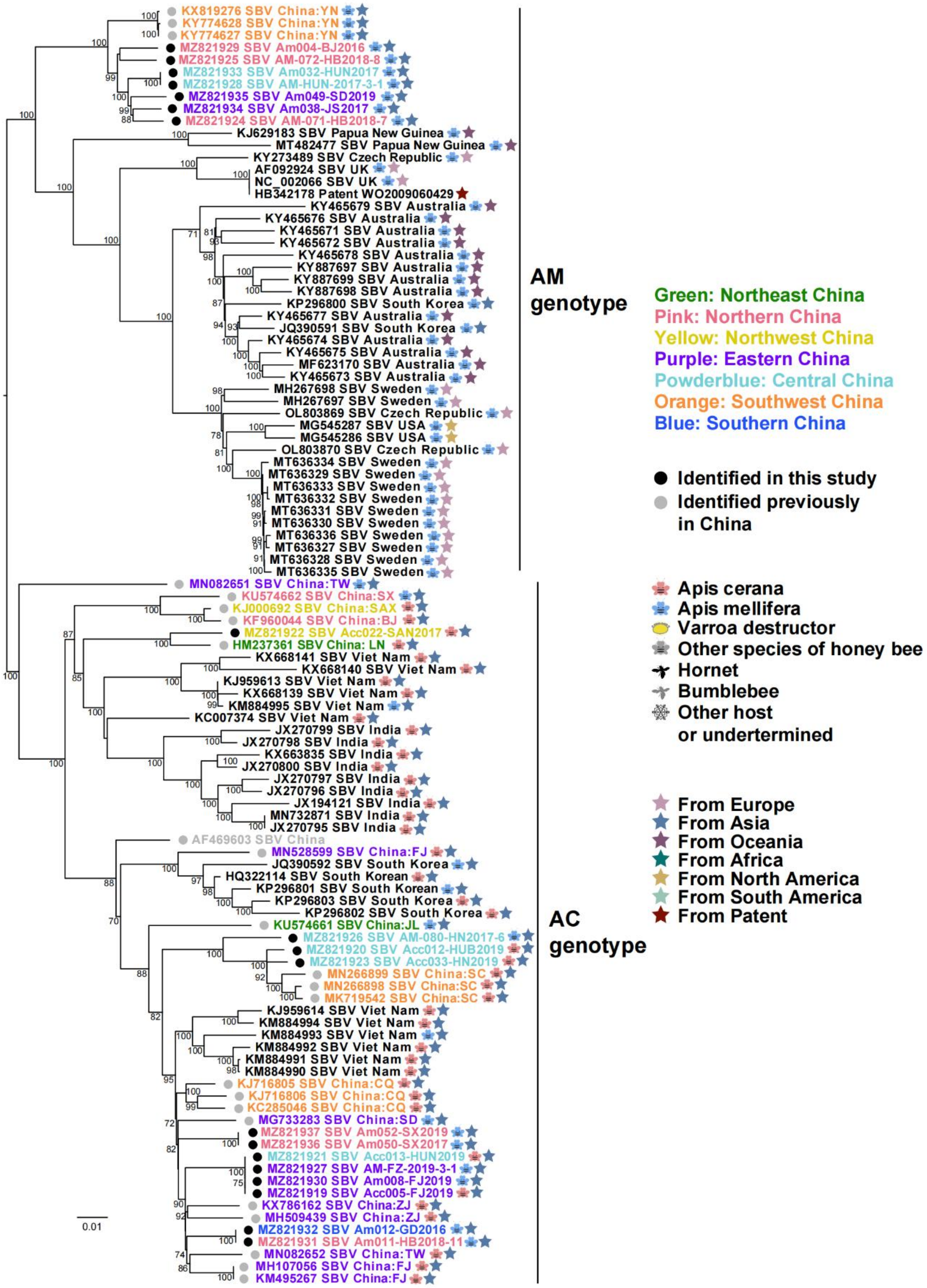

Figure S2. Maximum likelihood phylogeny of SBV. Figure legend follows Figure S1.

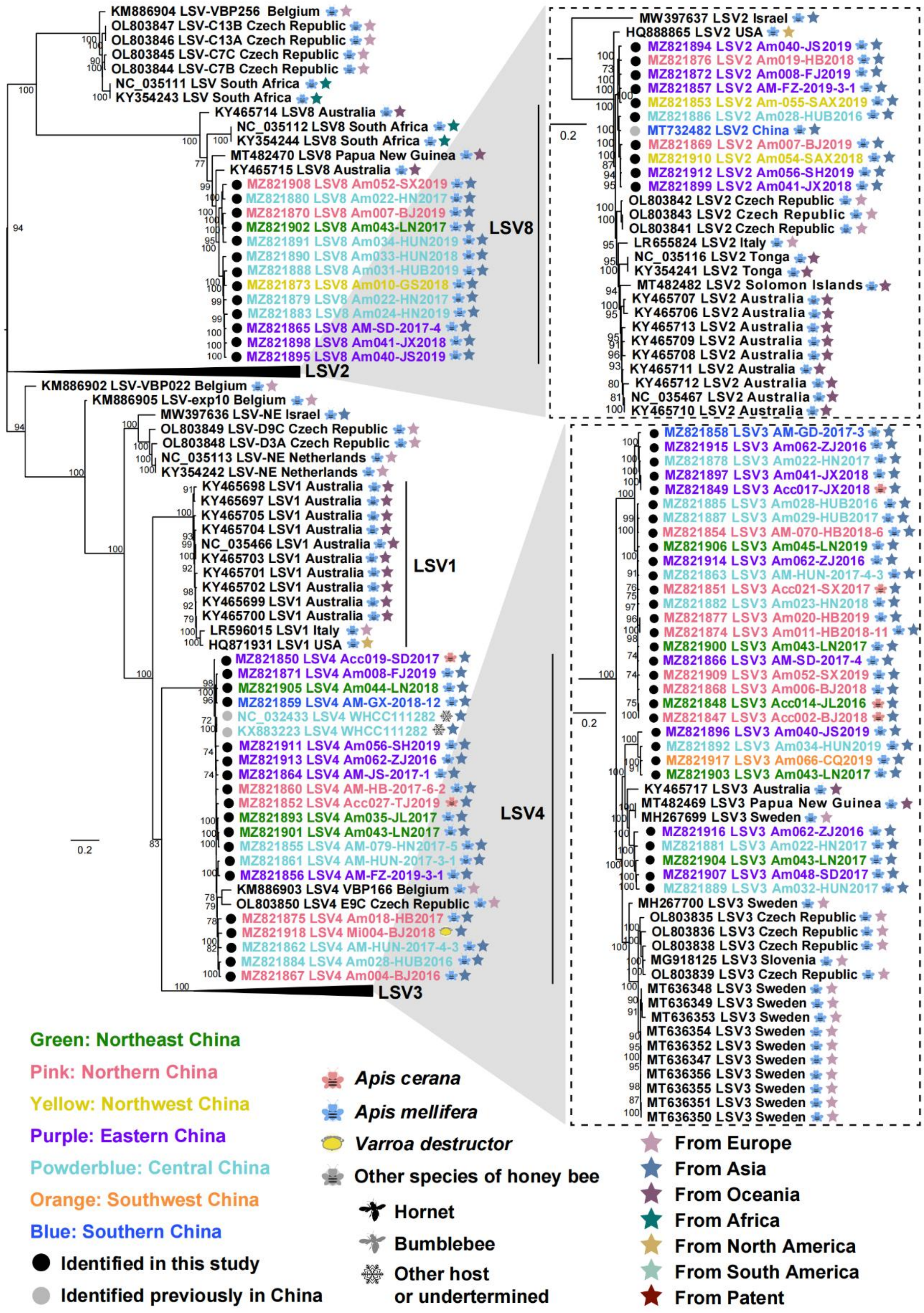

Figure S3. Maximum likelihood phylogeny of LSV. Figure legend follows Figure S1.

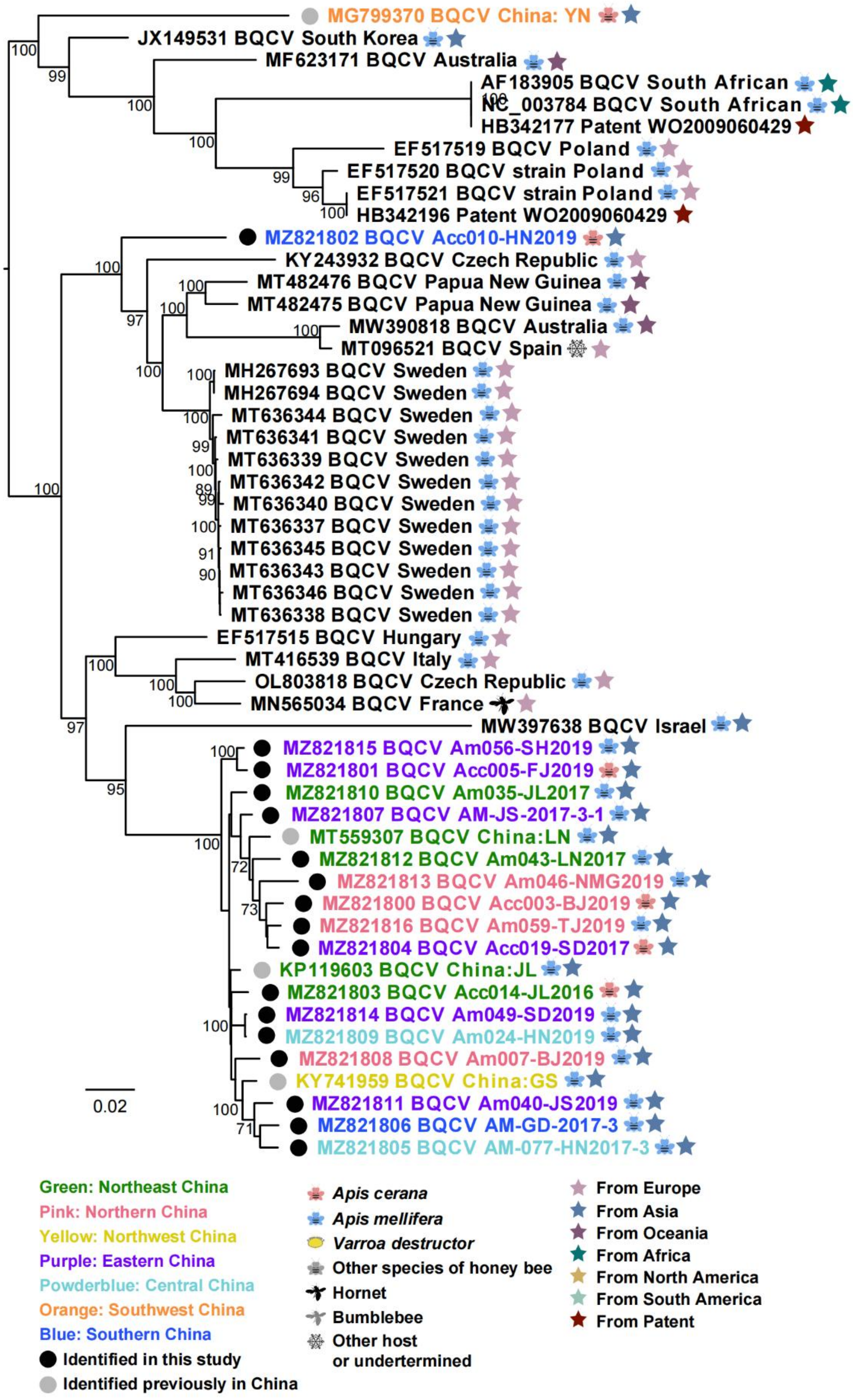

Figure S4. Maximum likelihood phylogeny of BQCV. Figure legend follows Figure S1.

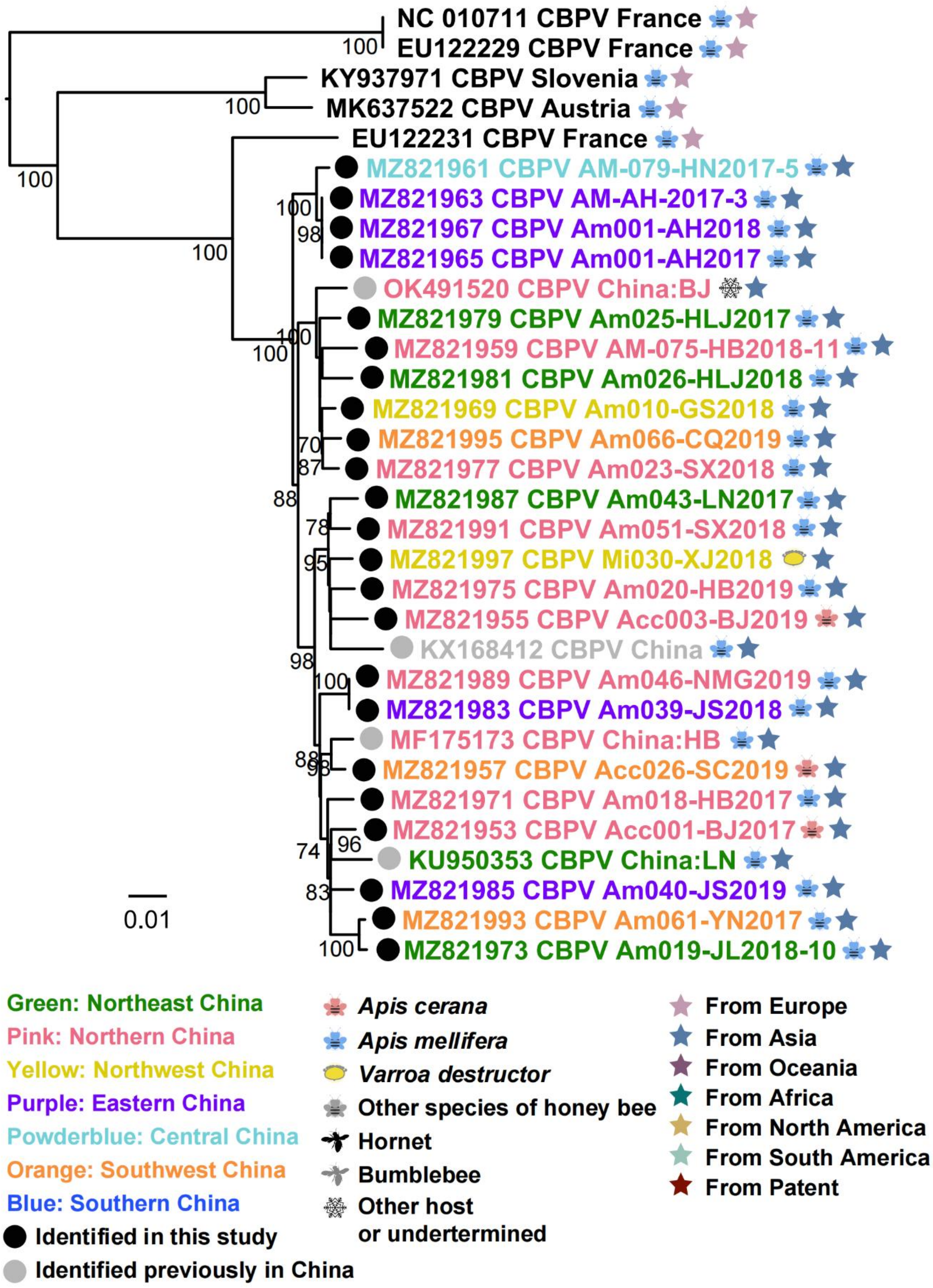

Figure S5. Maximum likelihood phylogeny of CBPV. Figure legend follows Figure S1.

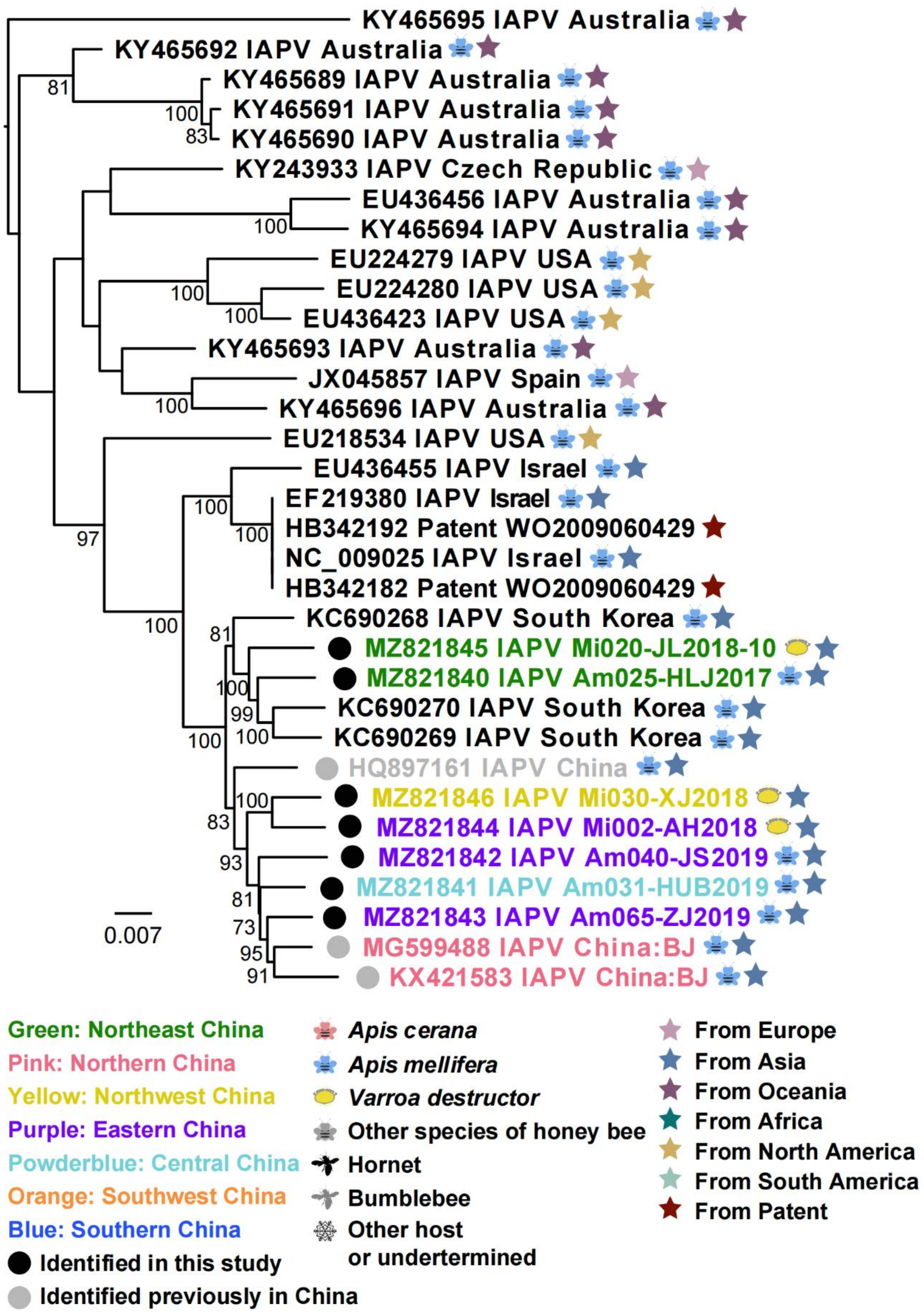

Figure S6. Maximum likelihood phylogeny of IAPV. Figure legend follows Figure S1.

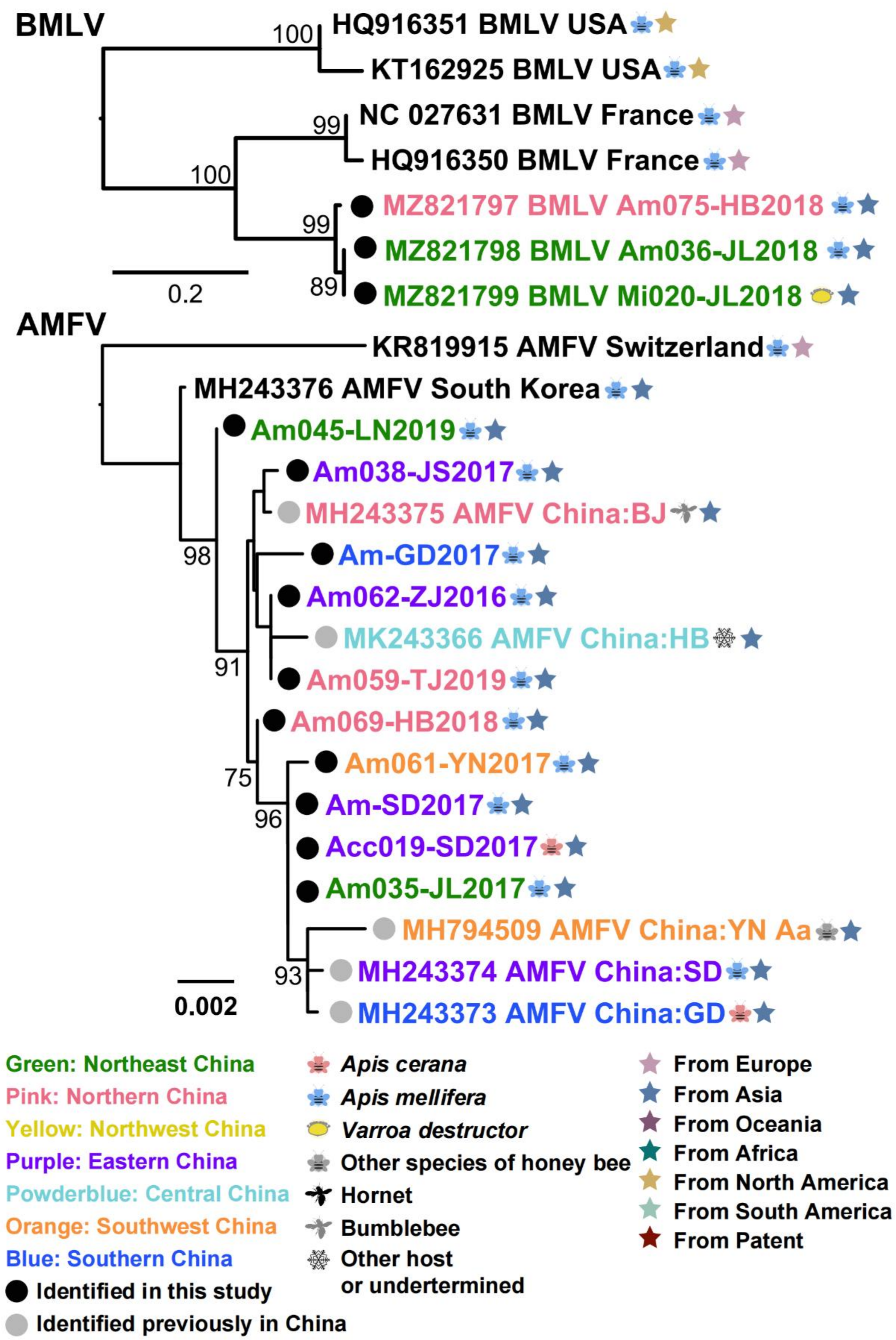

Figure S7. Maximum likelihood phylogeny of BMLV-AMFV. Figure legend follows Figure S1.

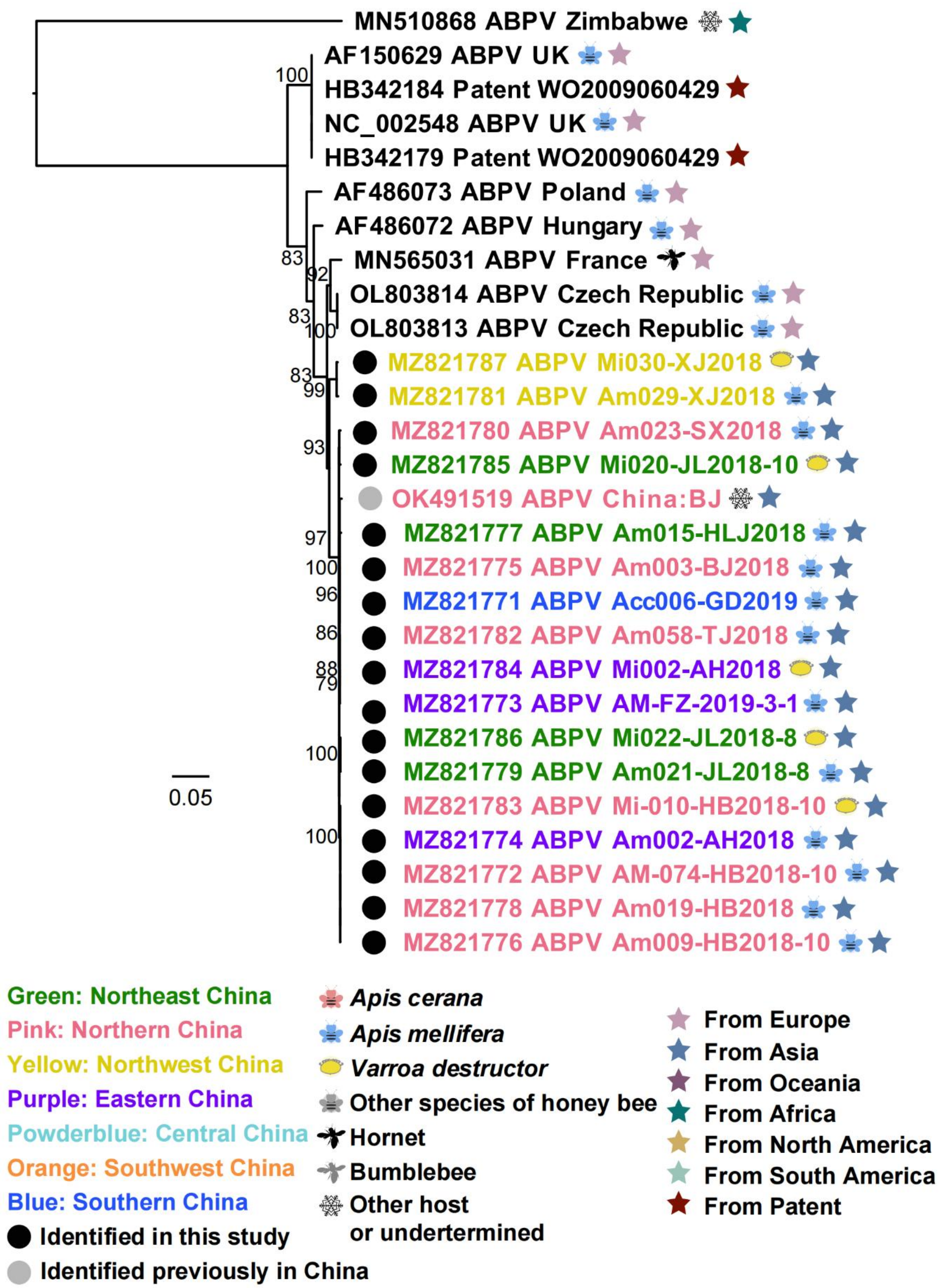

Figure S8. Maximum likelihood phylogeny of ABPV. Figure legend follows Figure S1.

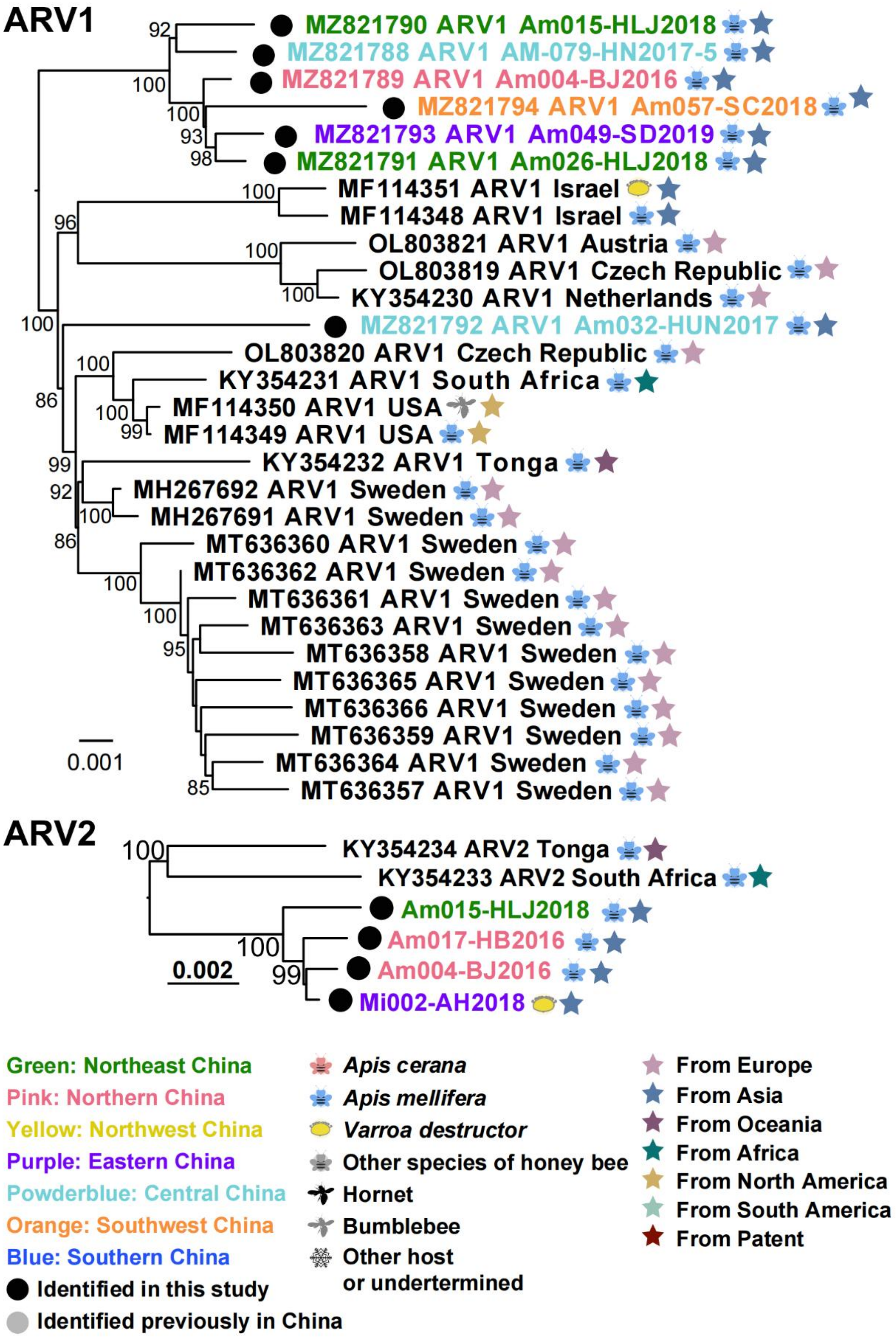

Figure S9. Maximum likelihood phylogeny of ARV1-ARV2. Figure legend follows Figure S1.

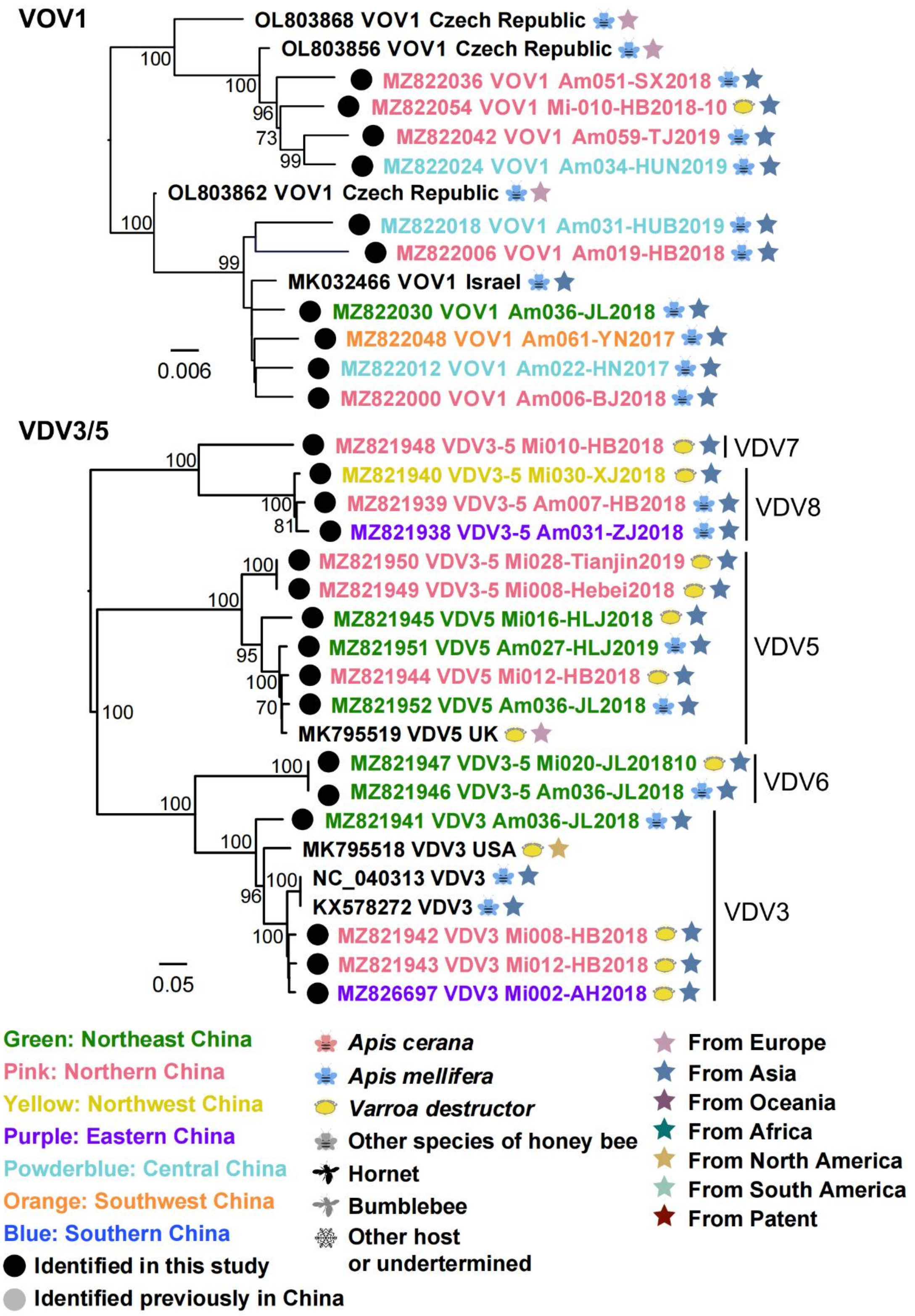

Figure S10. Maximum likelihood phylogeny of VOV1-VDV3/5. Figure legend follows Figure S1.

A

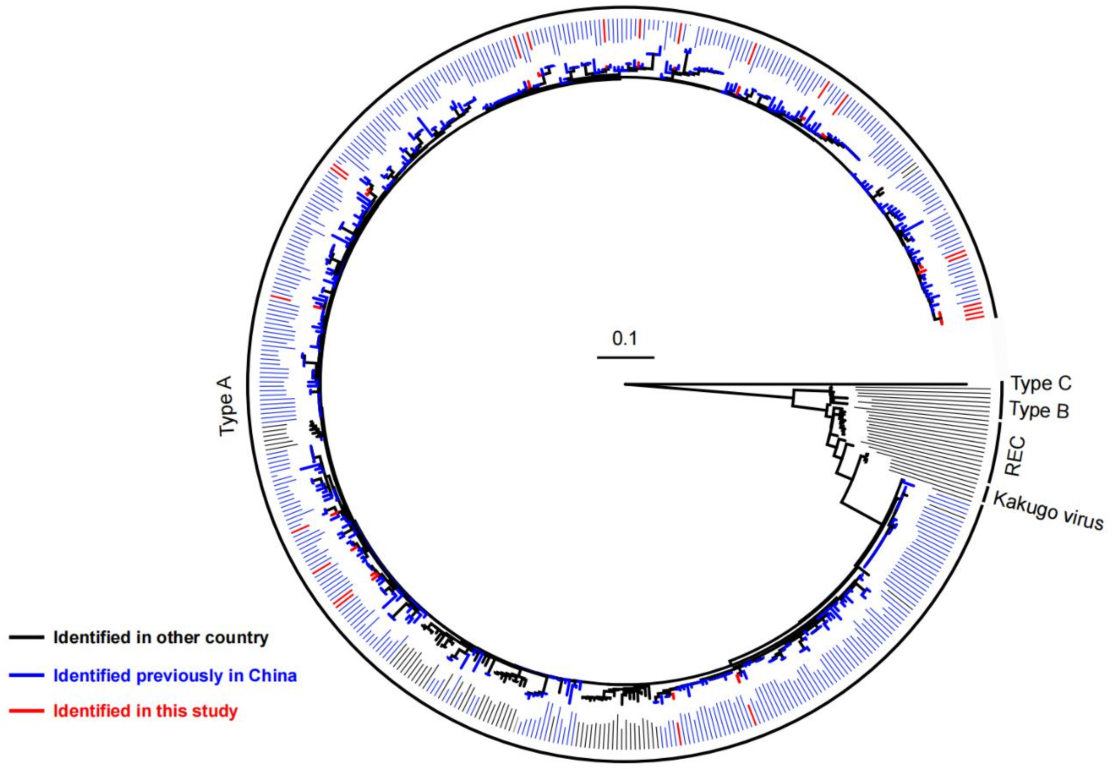

B

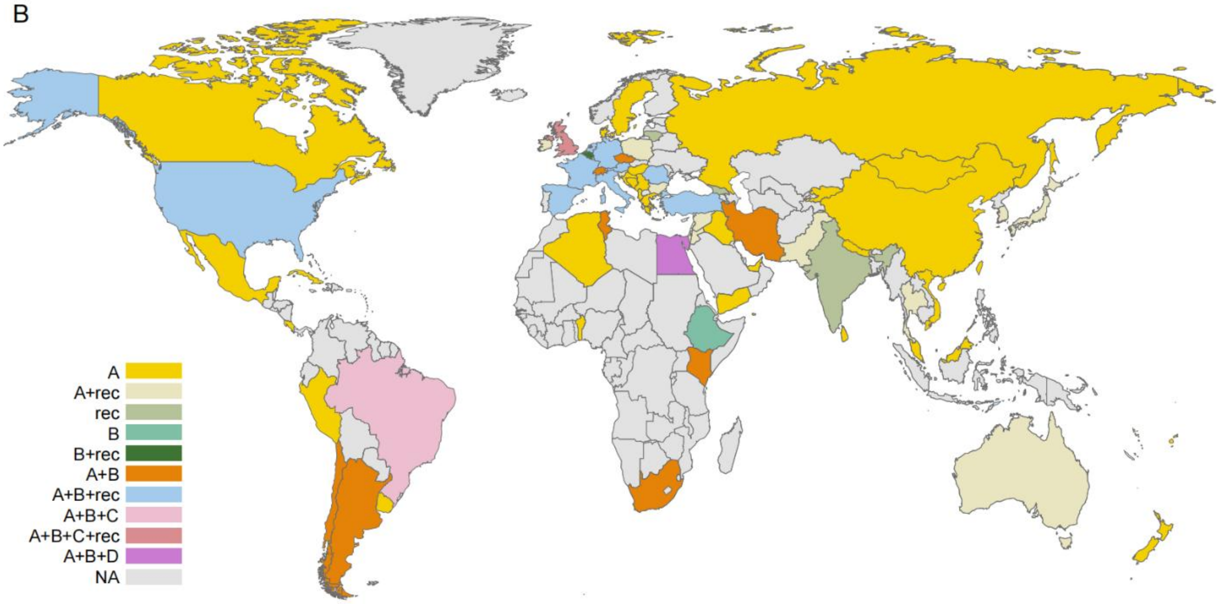

Figure S11. Intra-species diversity and global distribution of DWV. (A) The phylogenetic tree was estimated based all Chinese DWV strains (blue and red) and selected global reference sequences available on GenBank. Viruses identified in this study are colored red. (B) Global distribution of DWV. The map was constructed by combining global DWV data entries in GenBank with more country level information from reference papers.

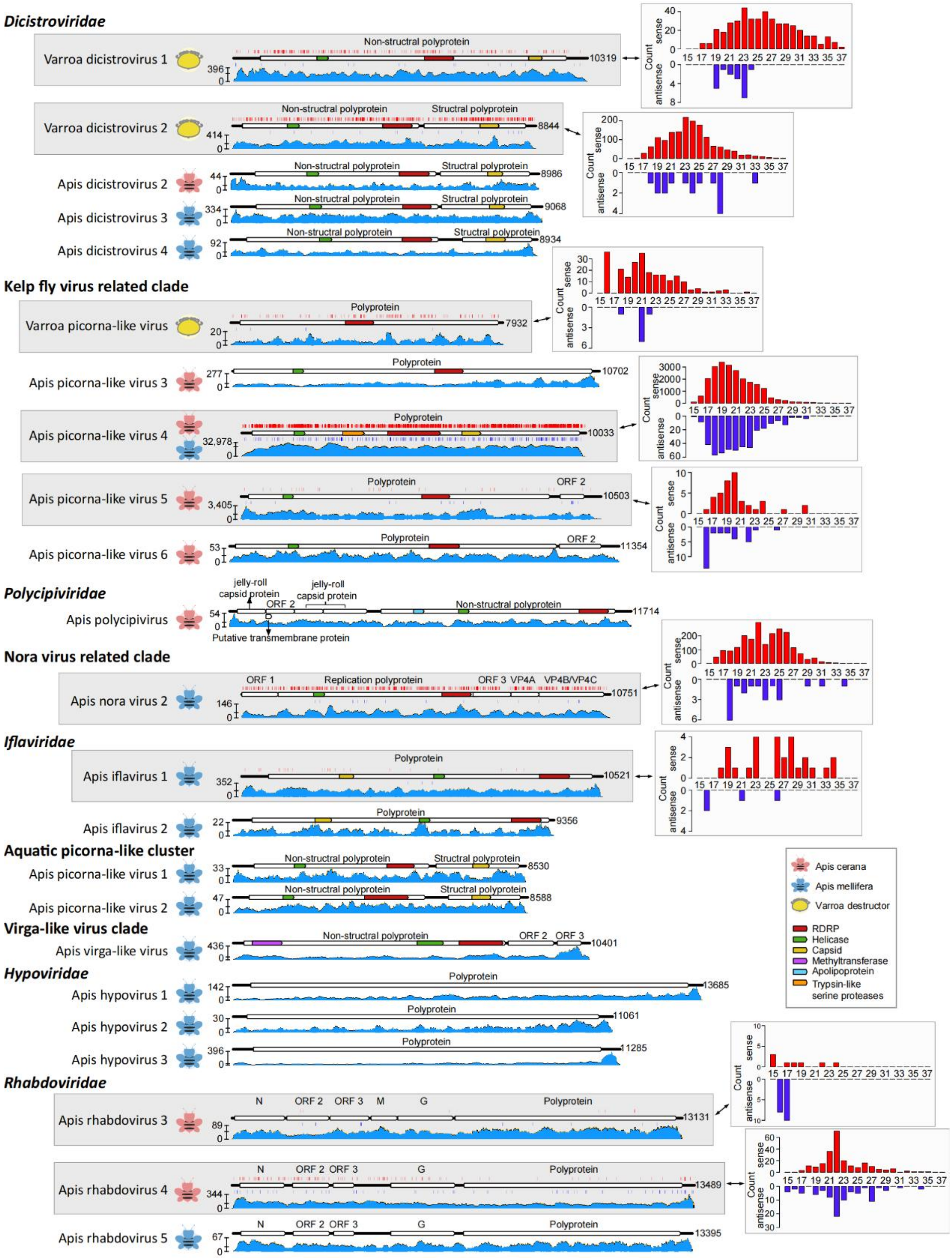

Figure S12. Summary of genomic features of the novel viruses identified in this study. The regions that encode major functional proteins or protein domains are labelled on each of the genomes. The host of each novel virus is shown to the left of the counterpart genome and read coverage is indicated below the genome. Size distributions (15-37 nt) of small RNA reads generated from positive viruses are shown right to the genome.

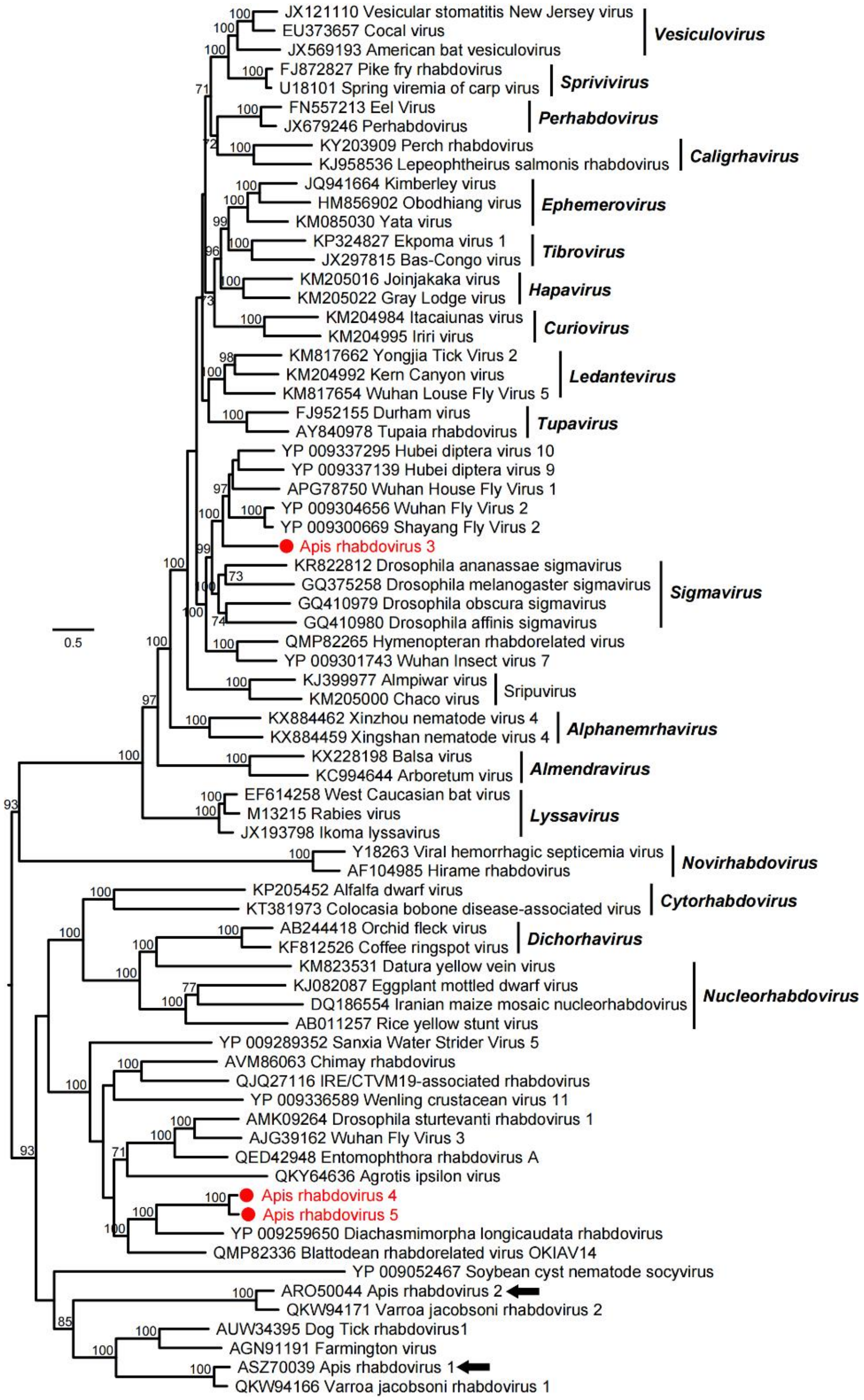

Figure S13. Maximum likelihood phylogeny of the rhabdoviruses. The newly described Apis rhabdoviruses 3, 4 and 5 are shown in red with red dots. Apis rhabdoviruses 1 and 2 that have been previously identified in honeybees are denoted with a solid black arrow.

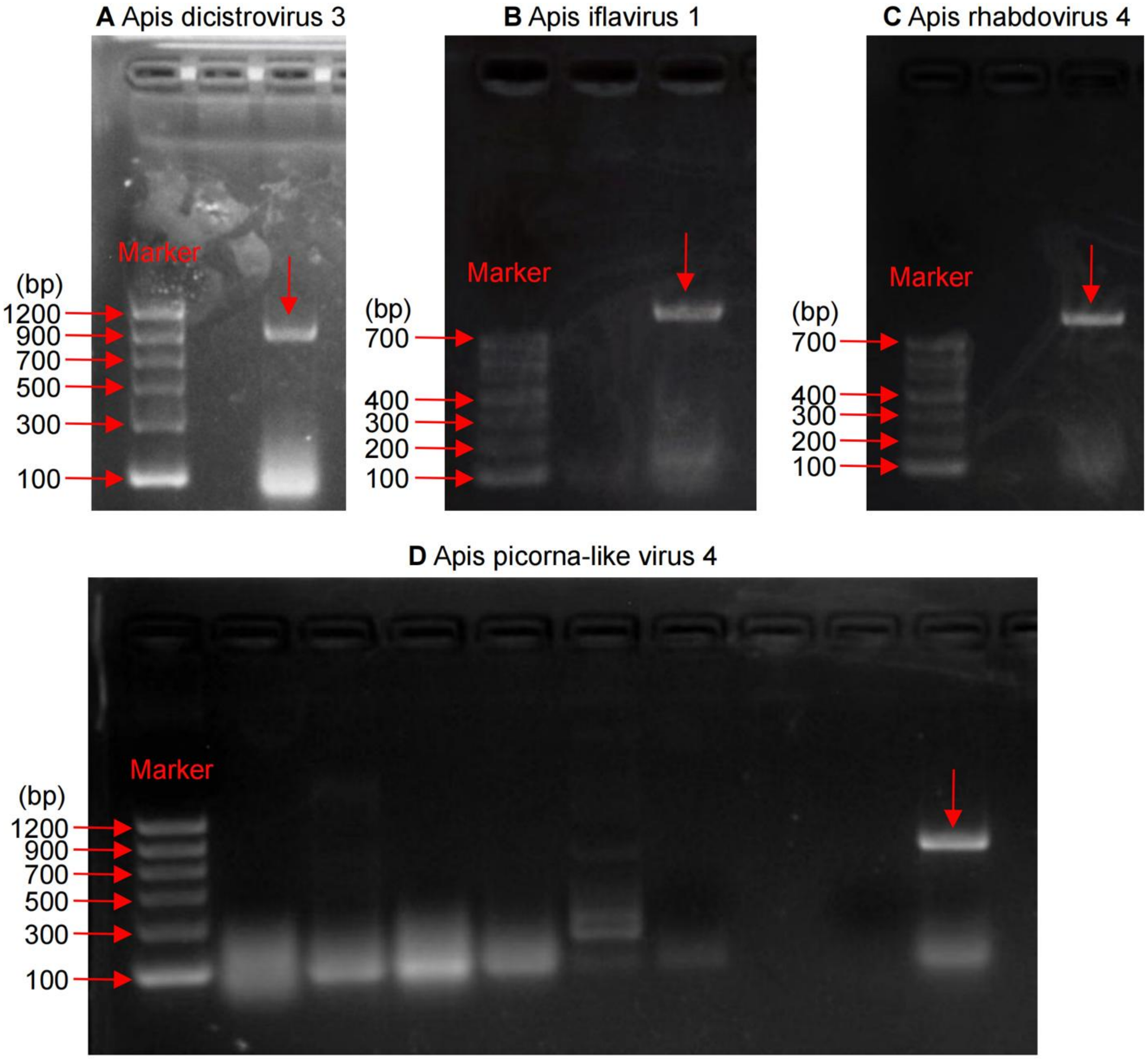

Figure S14. Tagged RT-PCR results on four viruses. (A) Apis dicistrovirus 3, (B) Apis iflavirus 1, (C) Apis rhabdovirus 4, (D) Apis picorna-like virus 4.

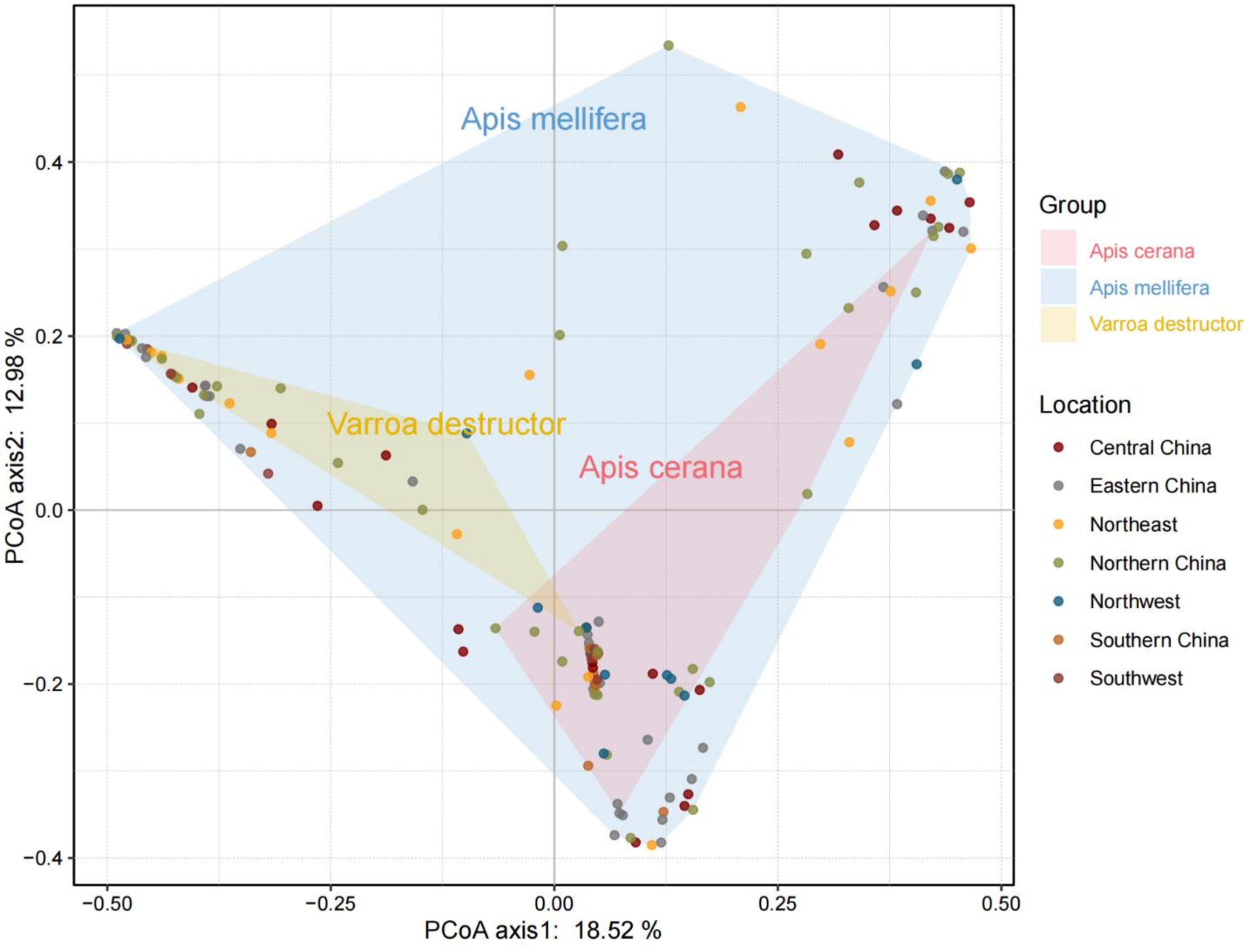

Figure S15. Beta diversity analysis of the viromic composition among libraries.
